# Communication Across Maternal Social Networks During England’s First National Lockdown and Its Association With Postnatal Depressive Symptoms

**DOI:** 10.3389/fpsyg.2021.648002

**Published:** 2021-05-11

**Authors:** Sarah Myers, Emily H. Emmott

**Affiliations:** ^1^UCL Anthropology, University College London, London, United Kingdom; ^2^BirthRites Independent Max Planck Research Group, Max Planck Institute for Evolutionary Anthropology, Leipzig, Germany

**Keywords:** postnatal depression, COVID-19, social distancing, lockdown, mothers, cooperative breeding, maternal social networks

## Abstract

Postnatal/postpartum depression (PND/PPD) had a pre-COVID-19 estimated prevalence ranging up to 23% in Europe, 33% in Australia, and 64% in America, and is detrimental to both mothers and their infants. Low social support is a key risk factor for developing PND. From an evolutionary perspective this is perhaps unsurprising, as humans evolved as cooperative childrearers, inherently reliant on social support to raise children. The coronavirus pandemic has created a situation in which support from social networks beyond the nuclear family is likely to be even more important to new mothers, as it poses risks and stresses for mothers to contend with; whilst at the same time, social distancing measures designed to limit transmission create unprecedented alterations to their access to such support. Using data from 162 mothers living in London with infants aged ≤6 months, we explore how communication with members of a mother’s social network related to her experience of postnatal depressive symptoms during the first “lockdown” in England. Levels of depressive symptoms, as assessed via the Edinburgh Postnatal Depression Scale, were high, with 47.5% of the participants meeting a ≥11 cut-off for PND. Quasi-Poisson regression modelling found that the number of network members seen in-person, and remote communication with a higher proportion of those not seen, was negatively associated with depressive symptoms; however, contact with a higher proportion of relatives was positively associated with symptoms, suggesting kin risked seeing mothers in need. Thematic qualitative analysis of open text responses found that mothers experienced a burden of constant mothering, inadequacy of virtual contact, and sadness and worries about lost social opportunities, while support from partners facilitated family bonding. While Western childrearing norms focus on intensive parenting, and fathers are key caregivers, our results highlight that it still “takes a village” to raise children in high-income populations and mothers are struggling in its absence.

## Introduction

Postnatal or postpartum depression (PND/PPD) is the term given to a bout of Major Depressive Disorder which has onset during pregnancy or within 4 weeks of birth (APA, 2013), though in practice it is applied to depression occurring within the first year from birth (Stowe et al., 2005; Halbreich and Karkun, 2006; Skalkidou et al., 2012). PND had a pre-COVID-19 estimated prevalence ranging up to 23% in Europe, 33% in Australia, and 64% in the United States (Arifin et al., 2018). PND predisposes mothers to future bouts of depression (Solomon et al., 2000), and becomes chronic in 38% of women (Vliegen et al., 2014).

While the evolutionary, ultimate function of depression is still under debate (Nettle, 2004; Hahn-Holbrook and Haselton, 2014; Myers et al., 2016, 2017; Hagen and Thornhill, 2017; Raison and Miller, 2017; Rantala et al., 2018), PND is associated with costs for both mothers and their children. For example, it inhibits a mother’s ability to care for herself and her infant (Downey and Coyne, 1990; Boath et al., 1998), and is associated with increased risks of a range of inflammation-related illnesses (Mykletun et al., 2009; Keicolt-Glaser and Glaser, 2002). PND is also associated with deficits in a range of children’s cognitive, social, and physical developmental outcomes (Cogill et al., 1986; Gelfand and Teti, 1990; Murray and Cooper, 1997; Beck, 1998; Wright et al., 2006), mediated in part by poorer mother-infant relations (Beck, 1995; Murray et al., 1996; Murray and Cooper, 1997; Coyl et al., 2002; Moehler et al., 2006; O’Hara and McCabe, 2013) which may last a life-time (Myers and Johns, 2018). Maternal mental health has increasingly been on the public health agenda due to the physiological and psychological consequences for mother-infant dyads, but also because of fiscal concerns – with the long-term costs of maternal mental health issues in the United Kingdom alone is estimated at £8.1 billion per 1-year cohort of births (Bauer et al., 2014). The full impact of the ongoing COVID-19 pandemic on maternal mental health is yet to be determined, but a picture of increased PND prevalence is rapidly emerging (e.g., see Davenport et al., 2020; Hessami et al., 2020; Spinola et al., 2020; Thayer and Gildner, 2020; Wu et al., 2020). As waves of COVID-19 continue to occur, it is crucial to understand how mothers are being impacted and what might mitigate their exposure to PND risk factors – here we take an evolutionarily informed focus on one such key risk factor, low social support (Beck, 2001; Yim et al., 2015; Doyle and Klein, 2020).

From an evolutionary perspective it is perhaps unsurprising that social support plays a role in maternal mental health, as humans evolved as cooperative childrearers where mothers require allomaternal support from kin and non-kin for successful reproduction (Hrdy, 1999; Hill et al., 2011; Dyble et al., 2015; Page et al., 2017; Emmott and Page, 2019). While the sources and nature of support vary across cultures, allomother [i.e., caregivers other than the mother (Hrdy, 1999)] presence and investments are generally associated with better maternal-child wellbeing (Sear et al., 2003; Gibson and Mace, 2005; Sear and Mace, 2008; Sear and Coall, 2011; Meehan et al., 2014). Mothers with infants are hypothesised to be particularly dependent on allomaternal support due to the high direct care needs of infants (such as prolonged carrying and high feeding frequency) which conflict with other activities (Hrdy, 1999); indeed, the postnatal period is often acknowledged in public health literature as a “vulnerable time” for mothers where they require high levels of support (Barlow, 2015; Johnston-Ataata et al., 2018). In Western contexts, despite the nuclear-family and intensive parenting norms (Faircloth, 2014; Sear, 2017) where fathers are key allomothers (Emmott and Mace, 2015; Emmott et al., 2020), public health literature shows that wider social support remains important for a range for postnatal health indices including maternal mental health (Crockenberg, 1981; Raj and Plichta, 1998; Beck, 2001; Kinsey et al., 2014; Yim et al., 2015; Emmott et al., 2020).

The COVID-19 pandemic has created a situation in which support from beyond the nuclear family is likely to be even more important to new mothers, as it poses actual and perceived health-related risks and stresses for mothers to contend with. At the same time, social distancing measures designed to limit viral transmission created unprecedented alterations to their access to such support. On the 23rd March 2020, England entered its first “national lockdown” following the increasing spread of COVID-19, where the government imposed social distancing measures requiring that individuals stay at home (unless exercising, shopping for food, or seeking medical attention), closed non-essential businesses and childcare facilities/schools, and banned public gatherings of more than two people. These measures remained in full force for almost 3 months until 14th June 2020, and likely impacted the social interactions of postnatal mothers in two primary ways: Firstly, by limiting in-person contact beyond the household, many women were no longer allowed to see their own mothers and other family members, as three-generation households containing young children are rare in the United Kingdom (Pilkauskas and Martinson, 2014). Family members, particularly maternal grandmothers, have been identified as important sources of childcare and domestic help in the United Kingdom (Emmott et al., 2020), meaning lockdown likely reduced the availability of practical support for mothers. Second, as antenatal classes and mother-baby groups were either cancelled or moved online during lockdown, potential interactions between new “mummy friends” were likely prevented, particularly for women giving birth after lockdown commenced. As female social networks often change in the perinatal period, with new supportive connections built with other women at a similar stage of pregnancy or motherhood (Nolan et al., 2012; Strange et al., 2014), this likely led to reduced social network connections among mothers, curtailing peer support. Although the cooperative childrearing literature typically focuses on female kin as key supporters on the grounds of inclusive fitness, who provides support appears to be flexible (Sear, 2021). New mothers benefit from reciprocal exchange of mothering-related support acts with non-related women at a similar stage of motherhood (Price et al., 2018; Finlayson et al., 2020). While there is limited data available on maternal social networks, mummy friends are likely to be particularly important in Western contexts where individuals frequently live long distances from kin.

Overall, lockdown led to a notably “unusual” childrearing environment even in Western contexts with strong nuclear family norms, limiting mothers access to allomother support beyond the nuclear family. Others have argued that lockdown measures would be highly detrimental for families with children (Arnot et al., 2020), particularly for mothers with infants (Doyle and Klein, 2020). Low social support, as noted, is known to increase the risk of PND, and social isolation also has strong links with depressive onset more generally (Lakey and Cronin, 2008). Therefore, it is crucial to understand the impact of social distancing measures on maternal mental health and the degree to which remote methods of communication are able to buffer against the detrimental consequences of reduced face-to-face contact. Here we explore how contact and communication within a mother’s social network relates to her experience of postnatal depressive symptoms during the first national lockdown in England. We focus on the experience of mothers in London – the initial epicentre of COVID-19 in England. This is due to our pre-existing project on maternal social networks in London, which was adjusted with the announcement of a national lockdown to investigate the impact of social distancing measures on maternal postnatal wellbeing.

## Research Aims

Here we take an exploratory approach to understand how social support networks existed during England’s national lockdown amongst London mothers, and their associations with postnatal depressive symptoms. Specifically, we explore: (Q1) Who did mothers keep in contact with during lockdown and how (in-person vs. remote communication)?; (Q2) Did characteristics of maternal social networks during lockdown vary by timing of birth in relation to lockdown?; (Q3) How did maternal social network characteristics and social communication during lockdown associate with self-reported depressive symptoms assessed via the Edinburgh Postnatal Depression Scale (EPDS; Cox et al., 1987)? We conduct a concurrent design mixed-method study (Leech and Onwuegbuzie, 2009) where quantitative and qualitative analyses were carried out at the same time. Using self-reported social network data, we quantitatively describe maternal support networks and analyse their associations with indicators of PND. In parallel, we thematically analyse open-text data from the survey to explore and understand the lived experiences of mothers with infants during England’s first lockdown. Both studies were preregistered before analysis (Quantitative study^1^ and Qualitative study)^2^ and minor deviations from our preregistered methods are outlined in the Supplementary Material 1. Finally, we synthesise the quantitative and qualitative findings to provide further insight.

## Data

### About the Survey

We use cross-sectional social network data from 162 London-based mothers with infants aged ≤6 months, collected in May–June 2020 (covering the first lockdown in England) using the formr online survey platform v.0.18.0 (Arslan et al., 2020). Postnatal depressive symptoms were assessed via self-report using the Edinburgh Postnatal Depression Scale (EPDS; Cox et al., 1987). The EPDS is the most commonly used screening tool for PND; it consists of ten items and gives a score out of 30, with a higher score indicating higher depressive symptoms. Participants were asked to report their personal social networks by listing everyone who is important to them, up to a maximum of 25 alters. For each alter, participants reported their age, gender, relationship, parental status, and age of their youngest child if relevant. They then reported who in their network they had seen in person, and who they had spoken to or messaged remotely (via phone, video calls, WhatsApp, Facebook, etc.) in the last few weeks. We also collected a range of demographic variables (See https://osf.io/k5whj/ for survey materials.) In total, the survey took around 15 min to complete.

Women were eligible to take part if they lived and gave birth in London, England, with a child aged 6 months or under at the time of the survey. We took an opportunistic approach to recruitment, advertising the study via social media platforms such as Facebook and Twitter (social networking sites). For Facebook, study adverts were posted on local mums/parents groups, local residents groups, and national baby groups. Studies have shown that social media survey recruitment can lead to an increased proportion of middle-class participants (Topolovec-Vranic and Natarajan, 2016). In order to track the age and educational background of women who were signing up (thereby allowing us to adjust recruitment strategy), eligible women were first required to register their interests on our study site; however, due to time constraints, all eligible women were eventually invited via email to complete the survey. Participants were given a £5 voucher upon completion of the survey as a token of thanks. Multiple entries were prevented using IP-address checks. Ethical approval for the survey was obtained from the UCL Research Ethics Committee (ref. 14733/002).

### Sample Characteristics

Mothers in our sample ranged in age from 19 to 47 years (mean 34.6, SD 4.2); half were first-time mothers (50.6%), while for 40.1% of women their infant was their second, 7.4% their third, and 1.9% their fourth child. The mean age of focal infant at the time of survey was 110 days (SD 56.6), with 115 infants born before 23rd March 2020, and 47 born after. Males comprised 54.3% of infants. The majority (53.7%) of births were reported to be uncomplicated, 34.6% associated with self-defined minor complications, and 11.7% major complications. The majority of infants were white (71.0%), 23.5% were of mixed ethnicity, and 5.6% were of other ethnicities. Only two participants reported not having a partner. Thirty-four percent reported an annual household income before tax of £0–75K, 19.8% £75,001–100K, and 34.6% over £100k (10 participants reported not knowing or preferring not to say, nine did not respond; see Supplementary Material for a detailed breakdown); the financial situation of the household had become worse during the pandemic for 29.0% of participants. The majority (87.0%) of participants were not socially isolating (i.e., staying at home and not going out because they or a household member had coronavirus symptoms or were vulnerable/at high risk) at the time of the survey. For a detailed breakdown of sample characteristics see Supplementary Material 2.

The full social network characteristics of participants can be seen in Table 1; on average, participants’ personal networks contained 11 alters, composed of 47.8% kin and 14.7% mummy friends (female non-kin with infants aged 18 months or under).

**TABLE 1:** Table shows a summary overall personal network characteristics of the sample, along with a summary of patterns of communication with network alters (*N* = 162).

Measure		Full sample			Time of birth in relation to 23rd March (before/after)		
		Range	Mean (SD)	Median	Range	Mean (SD)	Median
**Overall network characteristics**							
Total number of alters	*n*	1, 15	11.4 (6.8)	10	2, 25/1, 25	11.9 (6.9)/10.1 (6.3)	10/9
Kin	*n*	1, 15	4.6 (2.5)	4	1, 15/1, 14	4.7 (2.5)/4.5 (2.5)	4/4
Mummy friends	*n*	0, 10	1.9 (2.2)	1	0, 10/0, 9	2.2 (2.3)/1.3 (1.9)	1/1
**Seen in person in the last few weeks**							
Total number of alters	*n*	1, 14	3.9 (2.9)	3	1, 14/1, 11	3.9 (2.9)/4.0 (2.7)	3/3
	%	4.5, 100	40.9 (26.2)	33.3	4.5, 100/10.0, 100	38.7 (24.8)/46.4 (29.1)	33.3/40
Kin	*n*	1, 10	2.3 (1.6)	2	1, 10/1, 7	2.3 (1.6)/2.4 (1.7)	2/2
	%	10, 100	57.2 (31.6)	50	10, 100/14.3, 100	55.7 (31.0)/61.0 (33.2)	50/50
Mummy friends*	*n*	0, 5	0.5 (0.9)	0	0, 5/0, 4	0.6 (1.0)/0.4 (0.8)	0/0
	%	0.0, 100	31.2 (36.8)	20	0.0, 100/0.0, 100	30.9 (36.2)/32.1 (39.4)	20/5.6
**Communicated with remotely in the last few weeks**							
Total number of alters	*n*	1, 25	10.8 (6.4)	10	1, 25/1, 23	11.4 (6.6)/9.4 (5.6)	10/8
	%	50.0, 100	95.4 (8.9)	100	50, 100/61.1, 100	95.6 (8.7)/95.0 (9.4)	100/100
Kin	*n*	0, 15	4.4 (2.5)	4	0, 15/0, 9	4.5 (2.6)/4.1 (2.1)	4/4
	%	0.0, 100	94.5 (15.9)	100	0.0, 100/0.0, 100	95.2 (15.2)/92.9 (17.4)	100/100
Mummy friends*	*n*	0, 10	1.8 (2.1)	1	0, 10/0, 8	2.1 (2.2)/1.2 (1.8)	1/1
	%	42.9, 100	98.0 (9.1)	100	42.9, 100/88.9, 100	97.4 (10.4)/99.6 (2.2)	100/100
**Communicated remotely but not seen in the last few weeks**							
Total number of alters	*n*	0, 22	7.1 (5.3)	6	0, 22/0, 14	7.7 (5.6)/5.7 (4.0)	6/5
	%	0.0, 100	56.9 (25.8)	60	0, 100/0, 90	59.3 (24.5)/51.0 (28.2)	64.3/57.1
Kin	*n*	0, 11	2.3 (2.2)	2	0, 11/0, 6	2.4 (2.3)/1.9 (1.9)	2/2
	%	0.0, 100	42.3 (32.0)	50	0.0, 100/0.0, 100	44.1 (31.2)/37.9 (33.8)	50/33.3
Mummy friends*	*n*	0, 8	1.3 (1.6)	1	0, 8/0, 7	1.5 (1.8)/0.9 (1.5)	1/0
	%	0.0, 100	66.8 (37.7)	75	0.0, 100/0, 100	66.5 (37.4)/67.5 (39.2)	75/88.9

*Sample size for percentages is 107 as some participants reported no mummy friends.

**FIGURE 1:**
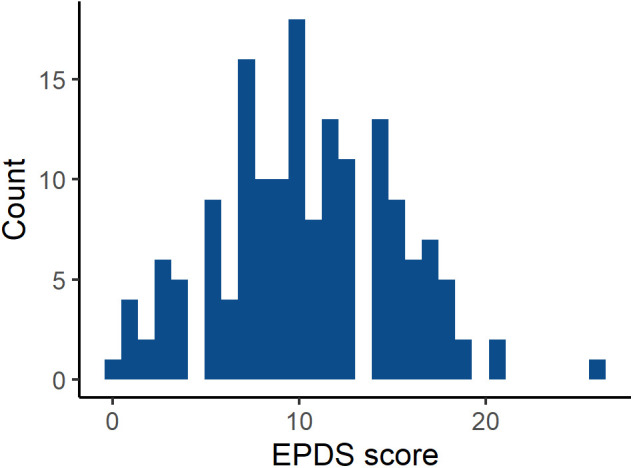
Histogram showing the distribution of Edinburgh Postnatal Depression Scale (EPDS) scores (*N* = 162).

Levels of depressive symptoms were high in our sample. Typically, EPDS scores in the general population are heavily skewed to the lower end of the scale (Alvarado et al., 2015; Coll et al., 2017; Martin and Redshaw, 2018; Smith-Nielsen et al., 2018; Boran et al., 2020); however, in our sample, they approximated a normal distribution with a mean of 10.4 (SD 4.7) (Figure 1 and Supplementary Figure 1). While comprehensive descriptive statistics of EPDS scores are rare in the literature, a pre-pandemic mean of 6.4 (SD 6.9) has been reported for English mothers at 3 months post-birth (Martin and Redshaw, 2018) and one of 7.2 (SD 4.4) among first-time Irish mothers at 6 weeks post-birth (Leahy-Warren et al., 2012). Our sample EPDS scores are therefore relatively high, and this elevation appears similar for women giving birth before 23rd March (mean 10.7, SD 4.9) and after (mean 9.9, SD 4.4), with little evidence of a correlation with days since birth (Supplementary Figure 3). A recent meta-analysis of EPDS usage recommends a cut-off of ≥11 to identify most women who would meet diagnostic criteria for PND and ≥13 for those with higher symptom levels (Levis et al., 2020); in our sample 47.5% and 34.6% of women, respectively, met these criteria for PND.

## Quantitative Analyses of Survey Data

### Quantitative Analyses Methods

Here we address the questions: (Q1) Who did mothers keep in contact with during lockdown and how (in-person vs. remote communication)?; (Q2) Did characteristics of maternal social networks during lockdown vary by timing of birth in relation to lockdown?; and (Q3) How did maternal social network characteristics and social communication during lockdown associate with Edinburgh Postnatal Depression Scale scores?

To address Q1, we quantitatively described social network characteristics and patterns of communication with network members. We also quantified the number and percentage of participants having either in-person or remote communication with the following key supporters: their own mother, their partner/infant’s father’s mother, a mummy friend. We also report *post hoc* exploration of in-person communication with kin by type (i.e., consanguineal vs. affinal), to shed light on findings in relation to Q3.

To address Q2, we examined differences in the aforementioned network characteristics by timing of birth, plotting the data and visually inspecting the distributions for changes in the pattern of social networks around the 23rd March when lockdown was imposed – which would be suggestive of a lockdown-specific effect. We took an exploratory approach rather than making predictions, because while the imposition of lockdown is a clearly defined event, it is possible that increasing public awareness led to behavioural alterations in the weeks before lockdown and/or any impacts of lockdown took time to develop. Secular trends may also be potentially attributable to lockdown, but with this data alone we are unable to disentangle them from infant age effects.

To address Q3, we used quasi-Poisson regression models to test for a relationship between social communication and EPDS scores. We anticipated seeing social contacts in person would have more of a protective effect on mental wellbeing than communicating with them remotely; thus, a first set of models assessed whether the number and/or percentage of (i) all alters, (ii) kin, and (iii) mummy friends seen in person (our independent variables) predicted EPDS score (our dependent variable). The *number of alters seen* reflects a mother’s in-person social network size during lockdown, capturing the number of people mothers have actually seen within their “network of important people” in the few weeks prior to the survey. While we do not have pre-pandemic data on maternal social networks, given the known relationship between closeness and in-person contact (e.g., Roberts and Dunbar, 2011), we interpret *percentage of alters seen* to reflect the extent to which mothers maintained in-person contact within their personal networks of important people during lockdown (i.e., of the important people in their lives, what proportion did mothers see in-person). To clarify, mothers could report a small *number of alters seen*, indicating a small lockdown in-person network, but report a high *percentage of alters seen*, indicating that they maintained in-person contact with a high proportion of important people in their lives. A second set of models were then used to assess whether having remote communication with those not seen, either as the absolute number or percentage of (i) all alters, (ii) kin, and (iii) mummy friends (our independent variables) predicted EPDS score (our dependent variable).

Model selection (outlined briefly below and in detail in the Supplementary Material 1) was performed between the following potential confounds influencing PND risk and/or social network characteristics to determine the control variables in our models: remote communication with social contacts, network size, partnership status, age of the mother, age of the infant, birth complications, infant sex, parity, has financial situation become worse since COVID-19, socially isolating at time of survey, infant’s ethnicity, household income, and time of birth in relation to lockdown.

Our model selection strategy was preregistered before exploratory analyses were conducted and stemmed from the base directed acyclic graphs (DAGs) in Supplementary Figures 2A,B, from which we used the R package dagitty (Textor et al., 2016) to select our control variables; the rationales for the relationships between our variables assumed in these DAGs are outlined in Supplementary Material 1. We ran a single control variable selection process using data relating to all alters, assuming the communication variants for (i) all alters, (ii) kin, and (iii) mummy friends share relationships with our potential confounds. From this starting point, we first updated the base DAGs based on the sample characteristics determined by exploratory analysis, and then assessed whether the implied conditional independencies from these updated DAGs were supported by the data (McElreath, 2020). Where independence was not supported, we updated our DAGs accordingly and repeated assessment of the newly implied conditional independencies until no updates were required (an overview of this process and the final DAGs can be found in the Supplementary Material 1; for full details see the R code available at https://osf.io/sr6d5/). We then selected the smallest minimally sufficient adjustment sets to adjust for in our models. We interpret the *number of alters seen* to reflect in-person network size during lockdown, and *percentage of alters seen* to reflect maintenance of in-person contact within personal networks during lockdown. We made no explicit prediction as to whether the *number* or the *percentage* of alters seen would be more important for predicting postnatal depressive symptoms; as a result, model selection produced two adjustment sets, one for when the independent variable of interest or exposure was the number of alters seen and one for when the exposure was the percentage of alters seen, equating to 12 models.

The variables retained in model selection were used as follows (for details of the variable derivation see the Supplementary Material 1): Independent variables: *in-person communication – number* was a continuous measure of the total number of (i) all alters, (ii) kin, or (iii) mummy friends seen in the last few weeks; *in-person communication – percentage* was a continuous measure of the percentage of (i) alters, (ii) kin, or (iii) mummy friends seen in the last few weeks; *remote communication but not seen – number* was a continuous measure of the total number of (i) alters, (ii) kin, or (iii) mummy friends communicated with remotely but not seen in the last few weeks; *remote communication but not seen – percentage* was a continuous measure of the percentage of (i) alters, (ii) kin, or (iii) mummy friends communicated with remotely but not seen in the last few weeks. Control variables: *age of infant*, measured in days, was used continuously; *age of mother*, measured in years, was used continuously; *parity* was used as a binary categorical variable of “1” (reference) vs. “2 or higher,” *household income* was used as a binary categorical variable of “£0–100K” (reference) or “over £100K,” *socially isolating* was used as a binary categorical variable of either “yes” or “no” (reference); *infant’s ethnicity* was used as a binary categorical variable of either “white” (reference) or “non-white.”

### Results of Quantitative Analyses

*Q1: Who did mothers keep in contact with during lockdown and how (in-person vs. remote communication)?*

On average, women had seen one family member other than their partner, three alters in total, and no mummy friends in the last few weeks (Table 1), while levels of remote communication were high across all categories of network alters. The majority (79.0%) of mothers in our sample reported their own mother as part of their personal network; of those, 49.2% had seen their mother in the last few weeks and 99.2% had communicated remotely with her (38.9 and 78.4% of all participants, respectively). Participants reported a median of 3 consanguineal kin (range 0–11) and 1 affine (range 0–6). While on average participants reported seeing the same number of kin across kin type (consanguineal and affinal: median 1, range 0–6), patterns within this differed. Forty-seven and a half percent of mothers had seen none of their own relatives, 17.9% had seen one, 19.1% had seen two, and 15.4% had seen three or more, with 25.3% seeing all their named consanguineal kin. On the other hand, 1.2% had seen no affines (the two single mothers in the sample), 8.0% had seen two affines, 4.3% had seen three or more, with the remaining 86.4% having seen one, i.e., only their partner, with 78.1% seeing all of their named affines. Only 19.8% of mothers listed their partner’s mother; of those 37.5% had seen her and 100% had communicated remotely (7.4 and 19.8% of all participants, respectively). The majority (66.0%) of participants reported having at least one mummy friend; of those, 54.2% had seen a mummy friend in the last few weeks and 100% had remote communication (35.8 and 66.0% of all participants, respectively). Weak (<0.3) to moderate (0.3 ≥ *r* < 0.7) positive correlations were found between the *number* and *percentage* of alters seen across categories, while correlations between the *number* and *percentage* remotely communicated with were weakly positive for all alters and kin and weakly negative for mummy friends (Supplementary Material 1).

*Q2: Did characteristics of maternal social networks during lockdown vary by timing of birth in relation to lockdown?*

**FIGURE 2:**
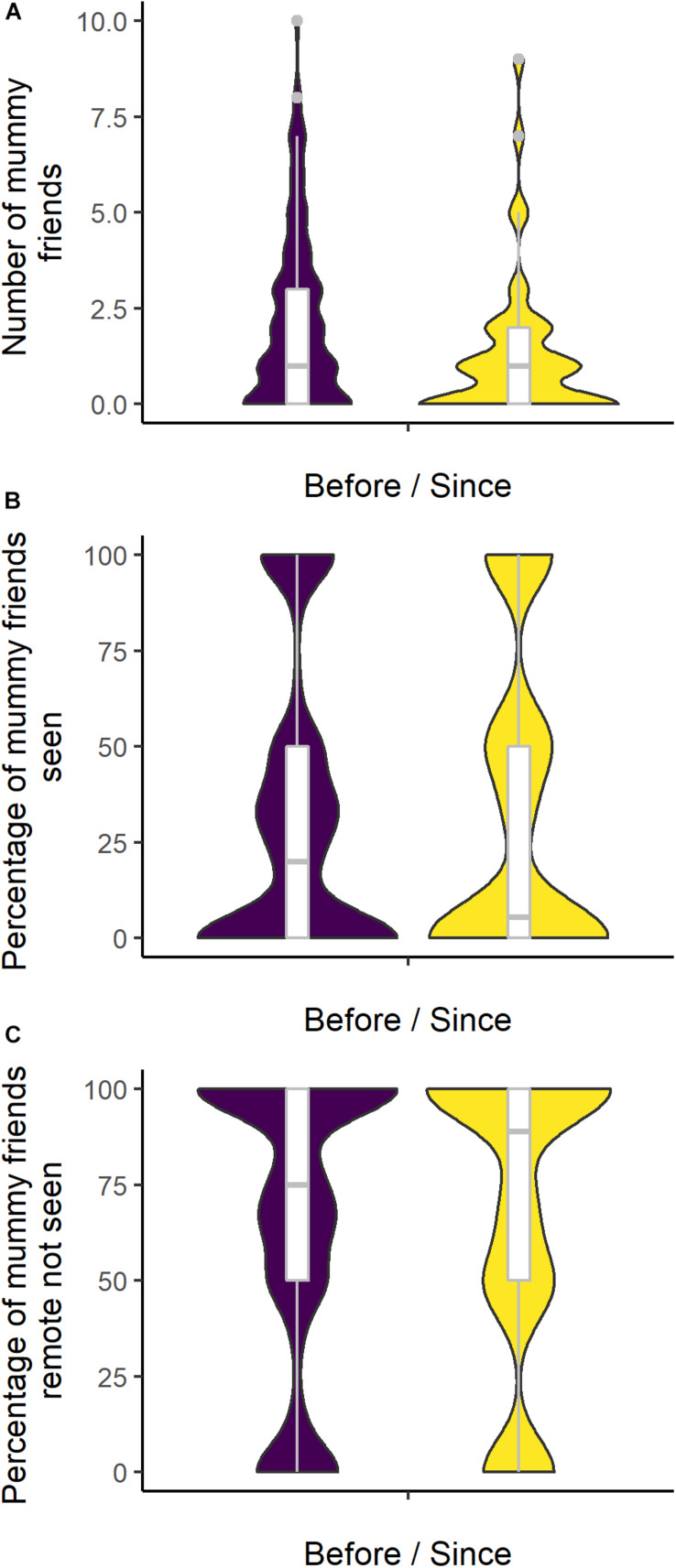
**(A–C)** Violin plots show the density distribution of network characteristics in relation to mummy friends by timing of birth, with in-set box plots showing the median and interquartile range [**(A)** *N* = 162; **(B,C)** *n* = 107].

We found only limited evidence of differences in the overall network characteristics of participants, dependent on whether they gave birth before or during lockdown (plots by date of birth can be seen in Supplementary Figures 4–14), with some indication that women giving birth on or after March 23rd had fewer mummy friends (Table 1 and Figure 2A). We found some indication that patterns of communication with mummy friends also differed between mothers giving birth since lockdown commenced to those giving birth before, with those giving birth since being less likely to have seen their mummy friends (Figure 2B) but more likely to have communicated with them remotely if they had not seen them (Figure 2C).

*Q3: How did maternal social network characteristics and social communication during lockdown associate with Edinburgh Postnatal Depression Scale scores?*

Here we present the quasi-Poisson regression models with the largest adjusted *R*^2^s (referred to as Models 1–3 for ease of interpretation – Table 2), while the remaining models performing better than chance can be seen in Supplementary Material 2; comparison of quasi-Kullback Information Criterion (QKIC) values indicated no clear “best” model (Burnham et al., 2011; Kim et al., 2013). As anticipated, the overall number of network alters seen in person over the last few weeks negatively predicted depressive symptoms, with the relative risk ratios (RRRs) ranging from 0.950 to 0.974 across models (Model 1 in Table 2, also see Supplementary Material 2). Once the number of alters seen was accounted for, contrary to expectations, point estimates for the proportion of the network seen had a positive association with depressive symptoms, a finding that appears to be driven by the effect of kin: The RRR for the percentage of network alters seen (Model 1) was 1.002 (CI 0.999, 1.005) and for kin (Model 2) 1.003 (CI 1.001, 1.006), and while the confidence intervals overlapped one for all alters, they narrowed when looking specifically at kin – this may be indicative of relatives being more likely to visit mothers in greater distress. *Post hoc* single variable models containing just the percentage of network alters seen found the direction of this effect to be the same when not controlling for number seen (see Supplementary Material 2). Finally, as anticipated, the greater the percentage of all alters communicated with remotely, if not seen in person, also appeared to be associated with lower depressive symptoms (Model 3) (RRR = 0.995, CI 0.999, 1.000); this pattern was repeated for the percentage of kin (RRR = 0.996, CI 0.991, 1.001), though with upper bound confidence intervals just overlapping one (see Supplementary Material 2). Models looking at mummy friends specifically did not perform better than chance (results not shown); this seems likely to be due to the widespread low levels of contact with other mothers with infants, with 55 participants reporting no mummy friends in their networks and on average mothers only reporting one. Note, our models varied in their performance and captured only limited variance in EPDS score, suggesting unaccounted for factors were playing an important role in maternal wellbeing, which is unsurprising.

**TABLE 2:** Results of quasi-Poisson regression models predicting depression symptoms as assessed via the Edinburgh Postnatal Depression Scale (EPDS) (*n* = 153).

Variables	Coef	SE	RRR	LCI	UCI
***Model 1: All alters*** –***in person communication (% as exposure)***					
(Intercept)	2.405	0.172	11.078	7.897	15.475
**In person communication (all alters)** – **%**	**0.002**	**0.002**	**1.002**	**0.999**	**1.005**
Age of infant	0.001	0.001	1.001	0.999	1.002
Infant’s ethnicity – non-white	0.044	0.082	1.045	0.889	1.225
Household income – over £100k	−0.054	0.075	0.948	0.817	1.098
**In person communication (all alters)** – **no.**	−**0.036**	**0.015**	**0.965**	**0.937**	**0.993**
Socially isolating – yes	0.003	0.114	1.003	0.799	1.248
Variance-function-based *R*^2^					0.121
Variance-function-based *R*^2^ – adjusted					0.085
***Model 2: Kin*** –***in person communication (% as exposure)***					
(Intercept)	2.178	0.173	8.833	6.275	12.384
**In person communication (kin)** – **%**	**0.003**	**0.001**	**1.003**	**1.001**	**1.006**
Age of infant	0.001	0.001	1.001	0.999	1.002
Infant’s ethnicity – non-white	0.067	0.081	1.069	0.911	1.250
Household income – over £100k	−0.040	0.076	0.961	0.828	1.114
**In person communication (kin)** – **no.**	−**0.026**	**0.027**	**0.975**	**0.924**	**1.026**
Socially isolating – yes	0.075	0.110	1.078	0.865	1.331
Variance-function-based *R*^2^					0.117
Variance-function-based *R*^2^ – adjusted					0.081
***Model 3: All alters*** –***remote communication but not seen (% as exposure)***					
(Intercept)	2.732	0.168	15.365	11.007	21.280
**Remote communication but not seen (all alters)** – **%**	−**0.005**	**0.002**	**0.995**	**0.991**	**1.000**
Age of infant	0.001	0.001	1.001	0.999	1.002
Household income – over £100k	−0.057	0.075	0.944	0.815	1.093
**In person communication (all alters)** – **no.**	−**0.051**	**0.017**	**0.950**	**0.918**	**0.983**
**Remote communication but not seen (all alters)** – **no.**	**0.013**	**0.011**	**1.013**	**0.992**	**1.034**
Socially isolating – yes	0.006	0.112	1.006	0.803	1.248
Variance-function-based *R*^2^					0.140
Variance-function-based *R*^2^ – adjusted					0.105

*Coef, coefficient; SE, standard error; RRR, relative risk ratio; LCI, 95% lower confidence interval; UCI, 95% upper confidence interval; no., number. Independent variables of interest are in bold.*

## Qualitative Analysis of Survey Data

### Qualitative Analysis Methods

To complement the quantitative analysis and help with overall inference, we conducted an inductive thematic analysis of open-text survey responses. In our survey, we asked three open-text questions: (Q1) “Is there anything you would like to share about how you feel the COVID-19 pandemic has affected your emotional relationship with your baby(ies)?” (Q2) “Is there anything you would like to share about how you feel the COVID-19 pandemic has affected your emotional wellbeing?” and (Q3) “Before you finish the survey, we would like to hear about anything else you feel is important to your experiences at this unusual and difficult time. If you have any other thoughts you would like to share, please do so below.” Questions therefore directed participants to elaborate on bonding with their baby(ies), their emotional wellbeing, and any other important matters they wished to raise. Of the 162 women who took part in the survey, 122 participants (75%) provided a response to one or more open-text questions, with 96, 82, and 77 participants responding to Q1, Q2, and Q3, respectively. Note, while these three questions were posed separately, the responses to these questions do not exist independently: For example, participants may refer back to their response in Q1 while responding to Q2, or decide to skip Q3 as they expressed all they wanted to in the earlier questions. This meant responses could not be analysed by question. In total, our data included 256 open-text responses with 61 words on average per response.

One of us (EE) conducted an inductive thematic analysis of open-text survey responses using NVivo v12. First, 96 open-text responses were coded in detail by EE and emerging themes were identified. Coding saturation was experienced early on at around 50 cases, implying relative similarity in the content of participant responses. EE then separately discussed emerging themes with SM and a subject-equivalent (new mother resident in London), and sought feedback on interpretation. Following discussion, EE amended the themes and coded all participant responses, with negative case analysis (i.e., specifically looking for participant responses which did not fit pre-specified themes). As a final validation step, as EE and SM do not have direct experiences of motherhood, EE and SM discussed findings with two mothers who had experienced lockdown with their infants to ensure findings were plausible. To minimise interpretive bias, the qualitative analysis method was designed and pre-registered in advance of any reading of participant responses (see text footnote 2) and was conducted parallel to the quantitative analyses by SM (i.e., results from quantitative findings were unknown at the time of qualitative analysis). Further information on the analysis process is outlined in the Supplementary Material 1.

The current method, combined with the characteristics of available data, was designed to identify underlying themes in the collective experiences of participants. As an exploratory, inductive design, we did not ask specific questions related to pre-existing ideas or hypotheses. Further, as open-text survey responses are relatively short, they are unlikely to provide a comprehensive description of maternal experiences during lockdown. This means that participants *not* raising certain experiences in their responses did not mean they lacked those experiences; therefore, our findings describe sample-level rather than individual-level characteristics. While we are unable to translate the findings to individual-level descriptors of maternal experience, our study nonetheless provides insight into the different types of experiences mothers in London encountered during the first national lockdown.

### Results of Qualitative Analysis

We identified four themes in our participant responses: One theme relating to the “benefits of lockdown” and three themes relating to the “costs of lockdown” (Table 3). These themes are not mutually exclusive and could have been experienced in different combinations, although the extent to which they overlap is unclear from our current study. The descriptions of each theme and example quotes are outlined below. More example quotes are available in the Supplementary Material 1.

**TABLE 3:** Summary of themes and key findings from our qualitative analysis.

	Main themes	Key findings
Benefits of lockdown	1. Enhancing bonding with baby within the nuclear family	Lockdown leading to uninterrupted time and “protection” of the nuclear family, leading to better bonding Facilitated by high levels of practical support from the partner
Costs of lockdown	2. The burden of constant mothering	Lack of practical support and childcare leading to the intensification of domestic and caregiving tasks Increased feelings of exhaustion and guilt
	3. Inadequacy of virtual contact	The lack of “incidental support” with virtual contact Lack on information transmission and affirmative support leading to low maternal confidence
	4. Lost opportunities	Inability to expand mother-infant social networks Feelings of sadness for lost experiences, for both mum and baby Worries about baby’s development due to lack of physical contact with others

#### Theme 1: Enhancing Bonding With Baby Within the Nuclear Family

For some women, lockdown gave them an unexpected opportunity to increase the “quality time” spent with the baby as a family, leading to perceived better bonding, improved relationships within the nuclear family, and positive emotional wellbeing. The stay-at-home and social distancing orders “protected” the nuclear family from visitors and non-essential commitments, leading to uninterrupted time and the ability to devote attention to the baby. Often, this was accompanied by high partner involvement, co-parenting, and practical support: As more people worked from home or were furloughed, partners were able to substitute maternal care allowing mothers to “take some time” and invest in themselves – such as catching up on sleep or exercise.

*“At first I was afraid that not being able to have any help might effect my relationship but in [fact] it has made it so strong because I’ve been with her all the time.…I have enjoyed the closeness and my son becoming closer to his baby sister.”*

*“The lockdown has been a big positive for my connection with the baby. I have been able to relax and enjoy her and give her my undivided attention versus [needing] to rush around, see people/host people etc. It has also been a great help having my partner home every day as it means most days I can get at least 1 h to myself which I use to exercise or catch up on sleep.…Also I think it has been an amazing positive on my partners ability to build a bond with the baby and co-parent.”*

#### Theme 2: The Burden of Constant Mothering

In stark contrast to the benefits of lockdown, many women also raised the burden of “constant mothering” due to the sudden severing of practical support. With no alloparents available to provide practical support and having to “do it alone,” mothers experienced the intensification of domestic and caregiving tasks. While the availability of partner support was not always clear, several women shared that their partners were not readily available to provide practical support due to work conflicts. This left mothers feeling exhausted and isolated, with no time to rest or recuperate, and some women reported feeling overwhelmed at the responsibility of looking after their child(ren) on their own. Particularly for mothers with multiple children, the lack of childcare and school closures meant women experienced competing demands for attention. This led women to experience guilt that they could not provide enough attention to their baby, with a few women expressing a sense of resentment toward their children and worsening family relationships. Overall, mothers were forced to devote their time to motherhood, and the ability to invest in other aspects of their lives including themselves and their relationships were severely restricted.

*“I think lockdown has made me feel like I’m not a person in my own right anymore, just a mum which is a feeling I had early on after my son was born but which disappeared when he was a few months old. Not having anyone else to hold him or help out a bit makes me feel it’s all me and it’s a lot of pressure which I can resent. I feel like I don’t have any time to rest.”*

*“Being able to just pass my baby round the loved ones would give me moments to enjoy without her, in the knowledge she was being stimulated and cared for by others. Without that it’s in a much smaller group of people to do that that leaves each of us far less time to focus on ourselves. I certainly miss being able to have people over and just hand them my baby so I can take 5 min to be [on] my own. I’ve only managed to be really alone once since she was born. Similarly, I’ve only once had 20 min with my partner without my baby being under our direct care since she was born that makes it hard for us to foster our connection. My mum does help but often we all sit together. I think without COVID I’d have more people offer to take her round the block and give us these moments of calm.”*

#### Theme 3: Inadequacy of Virtual Contact

During lockdown, many women relied on social media and remote communication methods such as WhatsApp and Zoom to keep in touch with others. A small number of mothers explicitly mentioned they have been able to maintain or even increase contact with others via remote communication methods, which served as important sources of support. However, many women commented on the inadequacy of virtual contact which led to a sense of isolation as well as worries and anxieties. Notably, mothers expressed that remote communication did not allow for unplanned and unsolicited support, where supporters incidentally identify maternal or infant needs and provide spontaneous reassurances or help. Without face-to-face contact, family, friends, peers, and health professionals could not “see the whole picture” regarding mothers and their infants, meaning mothers had to actively raise issues and seek support. This was particularly challenging for mothers who felt uncomfortable about asking for support or had low self-efficacy, leaving them with unmet support needs.

*“I’m the sort of person that doesn’t like to ask for help, and therefore relies on face to face contact to comfort and support me. I feel like the support I have has diminished now that I’m not able to have close contact with anyone outside my household…”*

Beyond support, the lack of face-to-face contact also acted as a barrier for information transmission to mothers from peers and health professionals. With fewer opportunities for “general chats” where women could raise minor questions or concerns, some women described their uncertainties around their parenting and baby’s development. Overall, this overlapped with increased anxiety and lower maternal confidence.

*“I joined a baby group and was hoping to get to meet up and share stories and learn how to be a mom from them and that [hasn’t happened]. I feel very like I am making it up as I go along and have no one to guide me as the health visitor can’t visit either. It is hard.”*

*“Your questions ask if I’ve had emotional support – yes, lots of communication and video calls. But during lockdown we were completely physically isolated which made us very stressed and anxious with no one to give practical advice on general parenting.”*

#### Theme 4: Lost Opportunities

Many mothers expressed their sense of sadness and grief in relation to lost opportunities with other people. In particular, women mourned the loss of “mummy friends,” as closing of parent-baby spaces and classes meant mothers were not able to establish new friendships for themselves and their babies as anticipated. Some mothers explained that this meant they had less access to peer support and advice. Further, several mothers mourned the loss of sharing “special moments” with family members, with specific concerns around the inability to establish bonds and connections between the baby and wider family.

*“This isn’t how I saw my experience with my first baby and sometimes it almost feels like grieving for an experience that we won’t get back. She is a beautiful baby and we are so lucky to have her. I wish I could share her and also actually discuss face to face with people what it is like being a mother – which they would be able to see physically.”*

*“You’re only a first time mum once, and I was really looking forward to this time and making new mum friends. I think I am most sad about missing out on that.”*

Many mothers believed these lost opportunities led to a sub-optimal developmental environment for the baby. In particular, mothers were worried that the lack of social experiences might negatively impact their baby’s long-term development. Mothers felt face-to-face contact with wider family and other babies were crucial for their development, which could not be adequately substituted by video calls. This was accompanied by a sense of guilt and worries about “over-attachment”.

*“I have a lot of worry about the developmental impact this will [have] on my son- I have already noticed he is more needy and although we regularly FaceTime them, I worry he won’t recognise my family members very well when lockdown is lifted. He seems to have developed physically and mentally so much in the last few months that It makes me worry more.”*

*“I feel so guilty that he will not be able to play with other children and worry how it will affect him.”*

## Synthesis of Quantitative and Qualitative Findings

Our sample of London mothers with infants exhibited high levels of depressive symptoms, with the mean self-reported EPDS score being 10.4, just 0.6 points below the recommended lower cut-off of 11 points to identify women who may meet the diagnostic criteria for PND (Levis et al., 2020). Forty-seven and a half percent of participants met the ≥11 cut-off for PND, and 35% of participants met the higher ≥13 cut-off for PND. The high levels of depressive symptoms in our sample were further supported by the themes arising in our open-text responses, with many women raising feeling stressed, anxious, and worried, as well as experiencing feelings of loneliness and sadness – overlapping with symptoms of PND (Cox et al., 1987; APA, 2013). Many women further reported they experienced a burden of “constant mothering” which left them physically and emotionally exhausted (Theme 2).

### Lockdown Network Size and Depressive Symptoms

Our quantitative results showed that, unsurprisingly, in-person contact during lockdown was low, with mothers typically seeing one family member other than their partner in the last few weeks. At the same time, levels of remote communication were high across maternal social networks. Contacting others in-person and remotely were both associated with lower EPDS scores, suggesting social network size during lockdown was associated with lower PND risk. Our qualitative findings highlight that in-person contact is important for practical support (Theme 1 and 2), as well as effective information transmission and emotional support (Theme 3), suggesting that mothers with higher levels of in-person contact may have been able to access more better-quality support, leading to lower depressive symptoms. However, our qualitative findings also revealed that the experience of remote communication was often felt as inadequate (Theme 3), somewhat conflicting with our quantitative findings. Taken together, our results perhaps reflect that, while remote communication is “not as good” as in-person contact, it could still bring some potential benefits; and remote contact may be better than no contact in mitigating depressive symptoms.

### Kin Support and Depressive Symptoms

While social network size during lockdown was associated with lower PND risk, proportionally higher in-person contact with kin network members - reflecting greater maintenance of their “important” kin network during lockdown - controlling for number of kin seen was associated with higher EPDS scores. Some of the cooperative childcare literature has noted differential impacts by kin type, with affinal kin sometimes found to be less beneficial and/or detrimental [most notably in relation to child outcomes (Sear and Mace, 2008)]. However, given the lack of variance in in-person contact with affinal kin, with most mothers only seeing their partner, our results appear to be driven by variations in contact with own kin. While our qualitative findings did not reveal the exact mechanism behind this association, the importance of practical support and in-person contact emerged as key components of maternal experience across multiple themes (Theme 1, 2, and 3). With this, one would expect in-person contact to associate with lower EPDS scores. A possible interpretation of our unexpected quantitative result is that, assuming in-person support is a more effective form of support (Theme 3), important kin members may have been more inclined to maintain in-person contact to support mothers experiencing PND symptoms. In-person contact did not only come with COVID-19 infection risk: it also risked fines (BBC News, 2020a) as well as public shaming and reputational damage (BBC News, 2020b). Kin members identified as important by mothers, given their relatedness and closeness, may have been more willing to take on these risks and potential costs to support mothers in need. However, it could also be that in-person communication with family members under socially distanced conditions served as a reminder of how much help mothers needed and/or were missing out on, thereby creating or exacerbating emotional distress. Indeed, mothers raised how lost opportunities for socialisation and support led to feelings of sadness (Theme 4).

### Peer Support and Depressive Symptoms

We found weak evidence that mothers who gave birth during lockdown had fewer “mummy friends” (i.e., female friends with young infants) compared to those who gave birth before. Further, we did not find evidence that contact with mummy friends was associated with lower depressive symptoms. This may be due to the lack of variation in the number of mummy friends reported by participants: On average, mothers in our sample reported just one mummy friend, with 55 participants reporting none. Nonetheless, our qualitative analysis revealed the potential importance of mummy friends, with mothers “mourning” the lost opportunities to make friends which could lead to maternal anxieties (Theme 4). Mothers also reported receiving lower levels of support and information from peers due to the inadequacy of virtual communication (Theme 3). Mothers indicated that in-person contact with peers is key for information transmission and affirmative support – which, in “non-COVID-19” times, would have helped women develop maternal capital in the form of parenting knowledge, skill, and confidence. It may be that mummy friends were not necessarily identified as “important people” within maternal social networks; alternatively, it may be that the pandemic has impeded the development of nascent mother-mother bonds in mothers giving birth prior to lockdown too, as well as their initial creation, leaving a wider cohort of women with few maternal social contacts. It is also worth noting that we used a narrow definition of mummy friends, limiting it to those currently with young children; it is more than likely mothers received support from friends at other stages of life too. Either way, peers may still be an important source of information and support – a resource mothers had limited access to during lockdown. Indeed, maternal support interventions in England and other developed populations often rely on organised peer support, with some evidence that this is associated with lower depressive symptoms (Leger and Letourneau, 2015).

## Discussion

Taking the quantitative and qualitative findings together, our results provide an in-depth description of maternal social networks during lockdown and its potential impact on depressive symptoms among the London mothers in our sample. The first national lockdown in England during the COVID-19 pandemic led to a childrearing environment which greatly minimised contact between households, curtailing access to support networks which typically provide allocare and other forms of social support (Emmott et al., 2020; Myers et al., 2021). In these arguably unusual times, our sample of London mothers with infants exhibited high levels of depressive symptoms with the mean EPDS score of 10.4 points. Forty-seven and a half percent of participants met the ≥11 cut-off for PND, and 35% of participants met the higher ≥13 cut-off for PND. This is notably higher than the pre-pandemic estimated PND prevalence of up to 23% in Europe (based on various measurement tools and cut-offs) (Arifin et al., 2018), but in line with other studies on maternal mental health during the COVID-19 pandemic. For example, recent studies conducted during the pandemic found 49% of mothers in a United Kingdom sample (Harrison et al., 2021), 33.2% in a Canadian sample (Cameron et al., 2020), and 23.6% in a Belgian sample (Ceulemans et al., 2020) met the ≥13 cut-off for EPDS scores, and 44% in an Italian sample met a ≥12 cut-off (Spinola et al., 2020). Multiple pre- vs. during-pandemic studies have also found increased prevalence of depressive symptoms (Wu et al., 2020; Zanardo et al., 2020); for example, a Canadian study found a jump from 15 to 40.7% of mothers meeting a ≥13 EPDS cut-off (Davenport et al., 2020). Combined with studies documenting elevated postnatal stress and anxiety (for example, see: Cameron et al., 2020; Ceulemans et al., 2020; Harrison et al., 2021), and more generalised measures of emotional wellbeing (Dib et al., 2020), there appears to be a broad picture of maternal emotional suffering on a wider scale than would be expected pre-pandemic.

It has been proposed that “depression is an adaptation designed to detect the opportunity costs of cooperative ventures and to subsequently bargain for increased benefits” (Hagen, 2003: 115). Under this framework, PND is argued to arise when the mother’s circumstances are such that withdrawing investment in their infant in the hope of eliciting support from others is the least bad option (Hagen, 2003). An alternative line of reasoning – the Pathogen Host Defence Hypothesis (PATHOS-D) – suggests that depression reflects a phenotypic suite of behavioural and physiological responses evolved to mitigate mortality risk linked to pathogens (Raison and Miller, 2013). Psychosocial stress is argued to have been predictive of wounding and subsequent infection in ancestral environments – where low social support and isolation is supposed to have increased attacks from predatory species or conspecifics – driving selection for a pre-emptive response. The bargaining hypothesis, along with the related psychic pain hypothesis (Hagen and Barrett, 2007), and arguments proposing PND evolved as a distress signal to elicit support (Crouch, 1999; Crouch, 2002), in particular from kin and the infant’s father (Rantala et al., 2018), would all anticipate increased rates of PND in the childrearing conditions created by the pandemic. The PATHOS-D would also predict elevated rates of PND resulting from psychosocial stress associated with lockdown. We do not consider our data to favour any one hypothesis in particular; current hypotheses regarding the evolution of PND and depression more generally are also not without their critics (Nettle, 2004; Myers et al., 2016, 2017; Rantala et al., 2018). Nonetheless, regardless of the evolutionary origins, postnatal depressive symptoms are undoubtedly an indicator of distress and if relatives were most likely to come to a mother’s aid, inclusive fitness would explain why. Needs-based kin altruism in the context of reproduction and childrearing has been reported elsewhere (Schaffnit and Sear, 2017; Page et al.,2019a,b), and our findings may suggest that a limited number of family members “took the risk” to provide practical support for mothers.

New mothers are obviously not the only people to experience significant reductions in their social contact, posing the question as to whether the findings here simply reflect a population-wide elevation in depressive symptomology; or do postnatal women constitute a specific risk group during the ongoing pandemic. We suggest the answer to both these questions is “yes.” There is a mounting body of work supporting the contention that the prevalence of depression symptoms has increased at the population level (Bueno-Notivol et al., 2021); for example, a survey of the general adult population in the United States found a three-fold increase compared to pre-pandemic levels (Ettman et al., 2020) and childhood depression data from the United Kingdom also suggests symptom elevation (Bignardi et al., 2020). However, evidence from the United Kingdom suggests that after an initial increase in symptoms in the run up to lockdown (Shevlin et al., 2020), depressive symptoms in the adult population declined from an elevated starting point across the first 20 weeks of lockdown, suggesting people adjusted (Fancourt et al., 2020). While our data cannot speak to other groups, given social support and social isolation are negatively associated with depressive symptoms across age groups in the general population in Western contexts (Lakey and Cronin, 2008; Gariépy et al., 2016), it seems reasonable to suggest that lessons from our quantitative findings may generalise beyond postnatal mothers. It is also clear that the impact of the COVID-19 pandemic is not equitable and there is widespread evidence that structural inequalities along such lines as socioeconomic position, race, and gender are creating differential burdens. We would encourage future studies examining the role of social networks in shaping depressive experience during the pandemic across other groups, to identify those most at risk and possible intervention strategies.

In the United Kingdom, cultural norms of intensive mothering arguably slowed down policy shifts to allow childcare support during periods of lockdown (Emmott et al., 2021), and there have been many reports of partners and other key supporters being prohibited from antenatal appointments and births, financial support packages have been geared toward the male workforce, and there has been widespread rhetoric from politicians and the popular press encouraging the gendered division of childcare responsibilities, all to the detriment of mothers (Emmott et al., 2021). Add to this parenting stresses which are arguably more common in the postnatal period – for instance, sleep deprivation, postpartum pain, the need to learn/relearn skills such as breastfeeding – and it appears reasonable to suggest that postnatal mothers are at particularly high risk of depression during the ongoing pandemic. However, there are at least two studies reporting a *decrease* in postnatal depressive risk during the pandemic; southern Israeli women giving birth in quarantine showed lower PND prevalence (Pariente et al., 2020), as did mothers of lower socioeconomic status (SES) in New York (Silverman et al., 2020). Rather than casting doubt on our findings, these studies highlight the contextually specific impact of social distancing measures; the authors of the Israeli study speculate that the mothers in their sample benefited from greater family support in this context (Pariente et al., 2020), while lower SES mothers in New York appear to have benefited from both not having to work themselves and increased childcare support from partners forced to stay at home (Silverman et al., 2020). These studies align with the finding from our qualitative results that some women experienced greater support from working-from-home partners than they might otherwise have done. Scelza and Hinde (2019) have recently argued that human evolution took place in “an adaptive sociocultural perinatal complex” typified by extensive social support for the mother-infant dyad, resulting from the energetic and physical demands of gestation, birth, breastfeeding, and the dependent state in which infants are born and the slow rates at which they develop. To protect maternal mental health, evidence suggests we should protect this perinatal complex, both during the ongoing COVID-19 pandemic and beyond.

### Limitations

Convenience sampling, predominantly recruiting via social media, leads to potential biases, particularly in relation to the degree to which our sample’s use of online technology for communication and support seeking is representative. Our participants were relatively homogenous in terms of ethnicity and family formation; thus, the extent to which these findings generalise to other mothers from London, the United Kingdom, and other high-income contexts is unknown. Further, the median wage in London in 2019 was £38,272 (Office for National Statistics, 2019), putting a two-person household at approximately £76.5K; only 34% of our sample had a household income before tax of between £0–75K. Low SES is a known risk factor for PND, and lower SES has been found to increase the risk of depression among adults in the United States during the pandemic; thus it is possible the rate depressive symptoms based on our sample were an underestimate of the actual rates in London (although see Silverman, et al., 2020).

Specifically relating to our quantitative study, the sample size of women giving birth during lockdown was small (*n* = 47), limiting the confidence in any apparent differences between maternal experience dependent on timing of birth before and during lockdown. Beyond the findings regarding communication with social network members, the confidence intervals for all other measures in our models are wide and overlap one, which is suggestive of a lack of statistical power. Maternal social networks were measured by asking participants to list “important people”; however, our qualitative results suggest contact with peers who are not necessarily important at an individual level may be a key aspect of maternal social networks, which we are unable to quantitatively test in our data. Our models captured only limited variance in depressive symptoms, suggesting unaccounted for factors were playing an important role in maternal wellbeing, which is unsurprising. Two important factors, known to be predictive of PND risk, which our models cannot speak to are a mother’s level of access to social support (Fellmeth et al., 2021) – both in terms of received and perceived practical and emotional support – and previous history of mental health issues (Spry et al., 2021); for a comprehensive review of other PND risk factors see Yim et al. (2015). We also have no data on whether participants had sought or were currently receiving medical attention for PND. Finally, the cross-sectional nature of our data means inferences of the direction of causation between communication and depressive symptoms are untestable; we also do not know the timing of any symptom onset or prior history of depression, which would help speak to the direction of causation.

In terms of our qualitative study, the relatively brief nature of open-text responses means that our findings are unlikely to capture the full range and nuance of maternal experiences during lockdown. Due to the open nature of the survey questions and the brevity of responses, our findings provide descriptions of various maternal experiences at sample-level. It is therefore unclear how wide-ranging these experiences were, and if or how these themes overlapped at an individual level.

## Conclusion

To paraphrase another study of maternal mental health during the COVID-19 pandemic (Davenport et al., 2020), mothers in London were not OK during England’s first lockdown, with a substantial number of women meeting the diagnostic criteria for PND. While Western childrearing norms focus on intensive parenting (Faircloth, 2014), our results highlight that it still “takes a village” to raise children in high-income populations. Several studies from Europe and the United States have found that maternal domestic work and childcare increased during the COVID-19 pandemic (Calarco et al., 2020; Del Boca et al., 2020; Prados and Zamarro, 2020; Zoch et al., 2020), suggesting that the burden of lockdown may have disproportionately impacted mothers. While our qualitative results suggest high partner involvement may have been associated with more positive experiences of lockdown, and a Canadian study found lockdown may have encouraged greater partner participation in domestic work and caregiving (Shafer et al., 2020), overall, our results indicate that adequate support within the household was either not available or not enough for many mothers in our sample.

As cooperative childrearers, the availability of extended support from beyond the nuclear family is crucial, and in our study mothers with communication with larger social networks during lockdown fared better in terms of maternal mental health. Since the time of our data collection, mothers in London have experienced two further periods of lockdown – one for approximately a month over November 2020 and the second, beginning on December 20th and only beginning to ease at the time of writing. Recognising the vulnerability of new parents, from the 2nd December, households in England with infants were allowed to form a “support bubble” and have in-person contact with one other household (Department of Health and Social Care, 2020), which may help alleviate the detrimental impact of lockdown on maternal mental health. However, in-person contact comes with infection risk, and we anticipate face-to-face contact across maternal social networks will remain low due to ongoing restrictions. Previous studies have found that online social contact is a valued source of social support for mothers (Archer and Kao, 2018; Price et al., 2018; Teaford et al., 2019). Remote communication could potentially be a solution, with our findings of lower depressive symptoms among mothers who had remote communication with a higher proportion of their personal network that they had not also seen in person. However, our qualitative findings suggest that seeking support and information may be more challenging via remote communication – several studies have also highlighted the costs of remote communication, including “Zoom fatigue” (Archer and Kao, 2018; Epstein, 2020). It is important, therefore, that the burden of seeking contact does not fall on the mother. Instead, encouraging people to virtually reach out to the mothers that they know may be a low-risk way of improving maternal mental health in high-income contexts, where most people have the means to do so.

## Data Availability Statement

The raw data supporting the conclusions of this article will be made available by the authors, without undue reservation.

## Ethics Statement

The studies involving human participants were reviewed and approved by the UCL Research Ethics Committee. The participants provided their written informed consent to participate in this study.

## Author Contributions

SM led the project, designed and managed the data collection, cleaned the data, and conducted the quantitative analysis. EE supported the data collection and conducted the qualitative analysis. SM and EE interpreted the quantitative and qualitative results, and wrote the manuscript. Both authors contributed to the article and approved the submitted version.

## Conflict of Interest

The authors declare that the research was conducted in the absence of any commercial or financial relationships that could be construed as a potential conflict of interest.

## Footnotes

1 https://osf.io/cse4a

2 https://osf.io/82bwj

## Supplementary Material

The Supplementary Material for this article can be found online at: https://www.frontiersin.org/articles/10.3389/fpsyg.2021.648002/full#supplementary-material. The legends for the Supplementary Figures can be found in Supplementary Material 1.

Table 1.docx ‘Supplementary Material 1’

Table 2.xlsx ‘Supplementary Material 2’

Image 1.TIFF ‘Supplementary Figure 1’

Image 2.TIFF ‘Supplementary Figure 2’

Image 3.TIFF ‘Supplementary Figure 3’

Image 4.TIFF ‘Supplementary Figure 4’

Image 5.TIFF ‘Supplementary Figure 5’

Image 6.TIFF ‘Supplementary Figure 6’

Image 7.TIFF ‘Supplementary Figure 7’

Image 8.TIFF ‘Supplementary Figure 8’

Image 9.TIFF ‘Supplementary Figure 9’

Image 10.TIFF ‘Supplementary Figure 10’

Image 11.TIFF ‘Supplementary Figure 11’

Image 12.TIFF ‘Supplementary Figure 12’

Image 13.TIFF ‘Supplementary Figure 13’

Image 14.TIFF ‘Supplementary Figure 14’

Image 15.TIFF ‘Supplementary Figure 15’
