# Supplementary material for: Communication Across Maternal Social Networks During England’s First National Lockdown and Its Association With Postnatal Depressive Symptoms
Source: Front Psychol. 2021 May 11;12:648002. doi: 10.3389/fpsyg.2021.648002 (PMC8144711; doi:10.3389/fpsyg.2021.648002)
Supplement: Table 1.docx — ‘Supplementary Material 1’ [file Table_1.docx]

Supplementary Information File 1

Contents

[Supplementary Information for Study 1 Quantitative Analyses 2](#_Toc70356240)

[Overview of Model Selection, Variable Derivation, and Deviations from the Preregistration 2](#_Toc70356241)

[Model selection 2](#_Toc70356242)

[Rationale underlying the paths between variables in the base directed acyclic graphs 2](#_Toc70356243)

[References for SI Table 1 4](#_Toc70356244)

[Variable derivation 5](#_Toc70356245)

[Deviations from Preregistration 5](#_Toc70356246)

[SI Table 2 Correlations Between the *Number* and *Percentage* of Alters Communicated With 5](#_Toc70356247)

[Supplementary Figure Legends 6](#_Toc70356248)

[Supplementary Information for Study 2 Qualitative Analysis 8](#_Toc70356249)

[Deviations from preregistered methods 8](#_Toc70356250)

[SI Table 3 Emerging topics from initial coding 8](#_Toc70356251)

[Notes from identification of preliminary themes 8](#_Toc70356252)

[SI Table 4 Additional example quotes for final themes 10](#_Toc70356253)

# Supplementary Information for Study 1 Quantitative Analyses

## Overview of Model Selection, Variable Derivation, and Deviations from the Preregistration

### Model selection

Our model selection strategy was preregistered before exploratory analysis was conducted and stemmed from the base directed acyclic graph (DAG) in SI Figure 2(A), from which we used the R package dagitty to select our control variables; the rationales for the relationships between our variables assumed in this DAG are outlined in SI Table 1. We ran a single control variable selection process, assuming the communication variants for i) all alters, ii) kin, and iii) mummy friends share relationships with our potential confounds. From this starting point, we first updated the base DAG based on the sample characteristics determined by exploratory analysis (removing parental status, due to lack of variance, and time of birth in relation to lockdown, due to little evidence a threshold effect in relation to network characteristics or EPDS score – see SI Figures 1, 3-14), and then assessed whether the implied conditional independencies from this updated DAG are supported by the data. Where independence was not supported, we updated our DAG accordingly and repeated assessment of the newly implied conditional independencies – this process was repeated until no updates were required, we then selected the smallest minimally sufficient adjustment sets to adjust for in our models.

We made no explicit prediction as to whether the *number* or the *percentage* of alters seen would be more important for predicting postnatal depressive symptoms, as a result the minimally sufficient adjustment sets differ dependent on whether number or percentage is chosen as the exposure variable, resulting in two models. Testing these models uncovered multicollinearity between network size, number of alters communicated with in person, and percentage of alters communicated with when looking at all alters and kin, thus we removed network size from our DAG and repeated the assessment of implied conditional independencies until no more updates were required (for the final DAG see SI Figure 15(A), for full details of all selection steps see the annotated R code available at <https://osf.io/sr6d5/>).

Second, following the same strategy, a further set of models were selected to assess whether having remote communication with those not seen, either the absolute number or percentage of the network, predicts EPDS score. The base DAG can be seen in SI Figure 2(B) and its rationale can be seen in SI Table 1. Network size and percentage of alters communicated with in person showed multicollinearity and were removed; the final DAG for these models can be seen in SI Figure 15(B) (for full details of all selection steps see the annotated R code available at <https://osf.io/sr6d5/>).

### Rationale underlying the paths between variables in the base directed acyclic graphs

SI Table 1. Rationale underlying the paths between variables in the base directed acyclic graphs; -> indicates the direction of inferred causality.

| **Paths in Base DAGs** | **Rationale** |
| --- | --- |
| Partnership status -> EPDS | Being a single mother is a risk factor for postnatal depression (Beck 2001) |
| Partnership status -> n/% of alters seen in person | Mothers with partners are likely to live with those partners and, therefore, to have seen someone in person; however, it is also possible that single mothers are more likely to need to reach out to network members beyond the household, increasing the proportion of their network seen in person |
| Partnership status -> income | The number of individuals contributing to household income will positively predict income |
| Partnership status -> impact of COVID-19 on income | The more individuals contributing to household income, the greater the likelihood one of their income’s will have been negatively impacted by COVID-19 |
| Income -> EPDS | Household income is an indicator of socioeconomic status (SES), being of low SES is a risk factor for postnatal depression (Beck 2001) |
| Impact of COVID-19 on income -> EPDS | A sudden reduction in income is likely to constitute a stressful life event, which is predictive of postnatal depression (Beck 2001) |
| Partnership status -> network size | Partners are likely to impact overall network size due to the addition of them and their own social connections (Gunnarsson and Cochran 1993) |
| Income -> impact of COVID-19 on income | Lower income jobs were more likely to be disbanded or furloughed (reducing salaries by 80%) than higher income jobs (Blundell et al. 2020) |
| Income -> network size | SES is known to predict network size (Cochran and Niego 2002) |
| Ethnicity -> income | Ethnicity is a predictor of income in the UK (Platt 2007) |
| Ethnicity -> impact of COVID-19 on income | The jobs disbanded or furloughed were disproportionately done by black and minority ethnic individuals (Blundell et al. 2020) |
| Ethnicity -> network size | Ethnicity has been found to predict network size in high income contexts (Cochran and Niego 2002) |
| Ethnicity -> n/% of alters seen in person | Differences in extended kin networks by ethnicity may alter the likelihood of seeing family members beyond the partner |
| n/% of alters seen in person -> n/% of alters communicated with remotely | It seems likely that there be a negative correlation between seeing alters and messaging them |
| Age of infant -> EPDS | Onset of postnatal depression peaks around 4 weeks postpartum (Stowe, Hostetter, Newport 2005); where swiftly diagnosed as treated, this is likely to result in a negative correlation with infant age and EPDS score. However, postnatal health services experienced widespread disruption during this time period (Jardine et al. 2020); it may be that women with older infants are more likely to display depressive symptoms, having been exposed to the stresses of motherhood during the pandemic for longer (Davenport et al. 2020). |
| Network size -> EPDS | To the extent that social network size is a proxy for available social support, then network size is likely to negatively predict EPDS score (Yim et al 2015) |
| Network size -> n/% of alters seen in person | Network size limits the number of alters that can be seen. If all mothers see a few members of their networks, but all are equally constrained in seeing people (as they were in theory by lockdown measures), then network size is likely to be negatively correlated with the percentage of their networks seen. |
| Network size -> n/% of alters communicated with remotely | Given the relative ubiquity of communication technology in the UK, network size is likely to positively correlate with number of alters communicated with remotely. Network size also seems likely to be negatively related to the percentage of alters communicated with. |
| Infant sex -> EPDS | Male infants have previously been found to be associated with heightened postnatal depression risk (Myers and Johns 2019) |
| Infant sex -> birth complications | Male infants are associated with increased risk of birth complications (Myers and Johns 2019) |
| Birth complications -> EPDS | Birth complications are associated with increased risk of postnatal depression (Myers and Johns 2019) |
| Birth complications -> socially isolating at the time of survey | Mothers who experienced birth complications may be more likely to be socially isolating due to increased vulnerability |
| Socially isolating at the time of survey -> EPDS | Mothers socially isolating due to increased vulnerability or there being COVID-19 symptoms among household members are likely to be under increased psychosocial stress, a risk factor for postnatal depression (Yim et al 2015) |
| Socially isolating at the time of survey -> n/% of alters seen in person | Mothers socially isolating are likely to have seen fewer alters and a lower percentage of the network |
| Socially isolating at the time of survey -> n/% of alters communicated with remotely | Mothers socially isolating may make more use of remote communication than those able to see alters in person |
| Parity -> EPDS | Given restrictions in access to nurseries, schools and informal childcare, mothers with additional children may be at increased risk of postnatal depression due to the stresses of additional childcare |
| Age of mother -> birth complications | Older mothers are at greater risk of birth complications (Cleary-Goldman et al. 2005) |
| Network size -> remote communication but not seen | If mothers are similarly restricted in seeing alters and only see household members, those with larger networks will have a greater number of unseen alters available to communicate with remotely |
| n/% of alters seen in person -> remote communication but not seen | The number of alters seen determine the number of remaining alters left to communicate with remotely |
| Socially isolating at time of survey -> remote communication but not seen | Those socially isolating may be in more need of social support and be more likely to message the alters they cannot see than mothers who have not seen alters for other reasons |

### References for SI Table 1

Beck CT. The effects of postpartum depression on child development: a meta-analysis. Archives of psychiatric nursing. 1998 Feb 1;12(1):12-20.

Blundell R, Costa Dias M, Joyce R, Xu X. COVID‐19 and Inequalities. Fiscal Studies. 2020 Jun;41(2):291-319.

Cleary-Goldman J, Malone FD, Vidaver J, Ball RH, Nyberg DA, Comstock CH, Saade GR, Eddleman KA, Klugman S, Dugoff L, Timor-Tritsch IE. Impact of maternal age on obstetric outcome. Obstetrics & Gynecology. 2005 May 1;105(5):983-90.

Cochran M, Niego S. Parenting and social networks. Handbook of Parenting Volume 4 Social Conditions and Applied Parenting. 2002 Mar 25;123-148.

Davenport, M.H., Meyer, S., Meah, V.L., Strynadka, M.C. and Khurana, R., 2020. Moms are not OK: COVID-19 and maternal mental health. *Frontiers in Global Women's Health*, *1*, p.1.

Gunnarsson L, Cochran M. The support networks of single parents: Sweden and the United States. The Social Networks of Parents and Their Children, Cambridge: Cambridge University Press, pp. 87-105. 1993.

Jardine J, Relph S, Magee LA, von Dadelszen P, Morris E, Ross‐Davie M, Draycott T, Khalil A. Maternity services in the UK during the coronavirus disease 2019 pandemic: a national survey of modifications to standard care. BJOG: An International Journal of Obstetrics & Gynaecology. 2020 Nov 5.

Myers S, Johns SE. Male infants and birth complications are associated with increased incidence of postnatal depression. Social Science & Medicine. 2019 Jan 1;220:56-64.

Platt L. Poverty and Ethnicity in the UK. Policy Press; 2007.

Stowe ZN, Hostetter AL, Newport DJ. The onset of postpartum depression: Implications for clinical screening in obstetrical and primary care. American journal of obstetrics and gynecology. 2005 Feb 1;192(2):522-6.

Yim IS, Stapleton LR, Guardino CM, Hahn-Holbrook J, Schetter CD. Biological and psychosocial predictors of postpartum depression: systematic review and call for integration. Annual review of clinical psychology. 2015 Mar 30;11.

### Variable derivation

Following exploratory analysis of the sample characteristics, the control variables persisting in the selected models requiring alteration from the raw data were derived as follows:

*Parity* – as pre-registered, number of biological children was collapsed into binary categorical variable of ‘1’ vs. ‘2 or higher’ due to the small number of participants at higher parities (parity 3 = 12, parity 4 = 3).

*Household income –* first those reporting ‘don’t know’ (n = 4) and ‘prefer not to say’ (n = 6) were added to the category with the largest sample size (i.e. the over £100k category). Next, the performance of the following categorisations were run in a Poisson version of the ‘All alters - in person communication (% as exposure)’ model and the model AICc’s compared: £0-75k, £75-100k, over 100k; £0-100k, over 100K; £0-75K, over 75K. AICc differences were minimal, nonetheless £0-100k, over 100K was selected having produced the smallest.

*Infant’s ethnicity –* was collapsed into a binary ‘white’ (reference) or ‘non-white’ categorical variable, on the grounds of small sample sizes across all non-white categories: South Asian/South Asian British = 4; Black/African/Caribbean/Black British = 1; East Asian/East Asian British = 4; Mixed/multiple ethnic groups = 38.

### Deviations from Preregistration

One – We preregistered (<https://osf.io/cse4a>) the use of Poisson regression models, prior to data exploration, on the grounds that Edinburgh Postnatal Depression Scale (EPDS) scores are integers and typically show a Poisson distribution with scores clustering at the lower end of the scale. However, in our sample EPDS scores approximate a normal distribution and the resulting Poisson models displayed over-dispersion. Fitting a negative binomial model did not address the issue with over-dispersion (see annotated R code), therefore we ran quasi-binomial models.

Two – Our base DAGs contained overall network size; however, the resulting models showed multicollinearity with communication variables and the decision was taken to remove network size in favour of leaning on the communication variables, as they were our independent variables of interest.

Three – Our original base DAG for the second set of models did not separate out the number and percentage of alters remotely communicated with but not seen; this was an oversight and we added this distinction to our base DAG before running the first round of testing implied conditional independencies.

## SI Table 2 Correlations Between the *Number* and *Percentage* of Alters Communicated With

| **Alter Category** | **Pearson’s r** | | |
| --- | --- | --- | --- |
|  | **In person** | **Remote** | **Remote communication but not seen** |
| **All alters** | 0.340 | 0.046 | 0.636 |
| **Kin** | 0.492 | 0.236 | 0.824 |
| **Mummy friends** | 0.636 | -0.070 | 0.485 |

## Supplementary Figure Legends

SI Figure 1. Q-Q plot of the distribution of Edinburgh Postnatal Depression Scale (EPDS) scores (n=162).

SI Figure 2(A-B). A) Base directed acyclic graph for assessing relationship between *seen in person* communication and Edinburgh Postnatal Depression Scale (EPDS) scores. Exposure = number (Seen_no) or percentage (Seen_per) of alters seen, outcome = EPDS. B) Base directed acyclic graph for assessing relationship between *speaking to/messaging remotely* with alters who were *not* seen in person and Edinburgh Postnatal Depression Scale (EPDS) scores. Exposure = remote communication but not seen (M_n_S), outcome = EPDS. Abbreviations: Income – household income; Income_worse – financial situation got worse since COVID-19; Network_size – overall network size; Partnership_status – partnership status; Soc_iso – socially isolating at time of survey; Ethnicity – infant’s ethnicity; Message_per – percentage of alters remotely communicated with; Message_no – number of alters remotely communicated with.

SI Figure 3. Scatterplot of the distribution of Edinburgh Postnatal Depression Scale (EPDS) scores by infant’s date of birth relative to lockdown beginning on March 23rd (n=162).

SI Figure 4. Scatterplot of the distribution of the total number of alters seen in person in the last few weeks by infant’s date of birth relative to lockdown beginning on March 23rd (n=162).

SI Figure 5. Scatterplot of the distribution of the percentage of alters seen in person in the last few weeks by infant’s date of birth relative to lockdown beginning on March 23rd (n=162).

SI Figure 6. Scatterplot of the distribution of the total number of alters communicated with remotely in the last few weeks by infant’s date of birth relative to lockdown beginning on March 23rd (n=162).

SI Figure 7. Scatterplot of the distribution of the total number of kin seen in person in the last few weeks by infant’s date of birth relative to lockdown beginning on March 23rd (n=162).

SI Figure 8. Scatterplot of the distribution of the percentage of kin seen in person in the last few weeks by infant’s date of birth relative to lockdown beginning on March 23rd (n=162).

SI Figure 9. Scatterplot of the distribution of the total number of kin communicated with remotely in the last few weeks by infant’s date of birth relative to lockdown beginning on March 23rd (n=162).

SI Figure 10. Scatterplot of the distribution of the percentage of kin communicated with remotely in the last few weeks by infant’s date of birth relative to lockdown beginning on March 23rd (n=162).

SI Figure 11. Scatterplot of the distribution of the total number of mummy friends seen in the last few weeks by infant’s date of birth relative to lockdown beginning on March 23rd (n=162).

SI Figure 12. Scatterplot of the distribution of the percentage of mummy friends seen in the last few weeks by infant’s date of birth relative to lockdown beginning on March 23rd (n=107).

SI Figure 13. Scatterplot of the distribution of the total number of mummy friends communicated with remotely in the last few weeks by infant’s date of birth relative to lockdown beginning on March 23rd (n=162).

SI Figure 14. Scatterplot of the distribution of the percentage of mummy friends communicated with remotely in the last few weeks by infant’s date of birth relative to lockdown beginning on March 23rd (n=107)

SI Figure 15 (A-B). A) Final directed acyclic graph for assessing relationship between *seen in person* communication and Edinburgh Postnatal Depression Scale (EPDS) scores. Exposure = number (Seen_no) or percentage (Seen_per) of alters seen, outcome = EPDS. B) Final directed acyclic graph for assessing relationship between *speaking to/messaging remotely* with alters who were *not* seen in person and Edinburgh Postnatal Depression Scale (EPDS) scores. Exposure = remote communication but not seen (M_n_S), outcome = EPDS. Abbreviations: Income – household income; Income_worse – financial situation got worse since COVID-19; Soc_iso – socially isolating at time of survey; Ethnicity – infant’s ethnicity; Message_per – percentage of alters remotely communicated with; Message_no – number of alters remotely communicated with.

# Supplementary Information for Study 2 Qualitative Analysis

## Deviations from preregistered methods

- One case of open text was identified to be from a test run of the survey, and was subsequently removed. This changed the sample size to 122.
- The RQDA package had been archived for the most recent version of R. Consequently, NVivo V12 was used for coding and thematic analysis.
- Coding saturation was experienced early into the initial detailed coding process, implying relative similarity in the content of open-text responses. To speed up the analysis process, detailed initial coding was conducted for 96 cases (instead of the full sample; n=122). The initial themes were then drafted after discussion between EE and SM. All 122 responses were then recoded into the preliminary themes, then finalised after further discussions with SM and two mothers who had infants during lockdown.

## SI Table 3 Emerging topics from initial coding

| Impact of lockdown: “winners and losers” | Emotions and feelings |
| --- | --- |
| - Feeling isolated vs Not having time alone - Devoting attention to baby vs Not enough time for baby - Strengthening relationships in household vs Damaging relationships in household | - worry and anxiety - sadness and depression - mourning and loss - monotonous, boring - pressure and responsibility - guilt for not providing optimal caregiving and environment for baby - gratitude and appreciation - "it could be worse" |

## Notes from identification of preliminary themes

**The following outline the emerging themes and notes after initial detailed coding, discussion with SM and discussion with one London mother, but before the second round of coding.**

*Theme 1: Enhancing bonding with baby*

- Severed social ties led to “protection” of the nuclear family from unnecessary visitors and social engagements/activities, leading to quality time between baby and members of the household. Overlapped with high practical support and caregiving from partner
- Better mother-infant bonding, but also infant bonding with partner and other children
- Improving relationship strength and quality within the household
- Comment (more for discussion?) Overlaps with idea of nuclear family norms and intensive parenting

*Theme 2: The burden of “constant mothering”*

- Severed social ties removed important practical support from kin and institutional alloparents (e.g., daycare and school). Amplified for those with older children due to lack of childcare, and those where partners not able to provide practical support due to employment (note, many women mentioned high emotional support from partner)
- Consequence: “Constant Mothering”
  - Intensification of domestic and caregiving tasks
  - Increased sense of pressure and responsibilities; “it’s a lot for one person”
  - No time for baby
  - No time for rest
  - Exhaustion, loneliness, sometime resentment towards children, worsening relationships within the nuclear family
- Comment (more for discussion)? Overlaps with idea of cooperative childrearing

*Theme 3: “Is this how it’s supposed to be?” Worries about mothering*

- Lack of information transmission and affirmative support from health professionals and other mothers/peers due to lack of face-to-face contact. Amplified for first-time mothers.
- Lead to anxiety about maternal competence and baby’s development
- Importance of face-to-face contact which cannot be replaced by virtual communication

*Theme 4: “How will this impact my baby?” Worries about development*

- Experienced social environment during lockdown very different from expected and the “norm”
- Worries about baby’s social development and long-term implications (socio-emotional development – somatic/intrinsic)
- Quite separately, worries about baby’s social network (social capital – extrasomatic/extrinsic)
- Worries accompanies by guilt about not providing optimal developmental environment; sadness about lack of opportunities

*Theme 5: Mourning lost opportunities*

- Stolen opportunities for new experiences, particularly around engaging with others:
  - Unable to make “mummy friends” and “baby friends” to expend social network
  - Unable to share experiences with friends and family
- Primarily accompanied by a sense of mourning and sadness, sometimes anger, that wider experiences were lost

## SI Table 4 Additional example quotes for final themes

| Category | Main Themes | Key Findings | Additional example quotes |
| --- | --- | --- | --- |
| Benefits of Lockdown | 1. Enhancing bonding with baby within the nuclear family | - Lockdown leading to uninterrupted time and “protection” of the nuclear family, leading to better bonding - Facilitated by high levels of practical support from the partner | “I feel the lack of social and work engagements has led to a relaxed routine at home. We have been able to respond to our babies hunger and sleep cues immediately as he has our full attention most of the time, and (whether or not this is a direct result) he is a contented happy baby who has organically developed a textbook routine and sleeps through the night!”  “I think that being at home under lockdown has helped us all bond as a family of 4. It has been helpful to have my partner home and I have had a bit more time to exercise etc than I think I would have had otherwise.”  “Having my partner around for more than his 2 weeks paternity has helped me to feel emotionally supported and get more sleep than I would have done, which has had a positive effect on my emotional well-being and therefor my relationship with my baby.”    “Although it has been a real shame that family and friends have been unable to visit/ have cuddles, I feel pleased to have had lots of uninterrupted time with my baby in these early weeks and to get to know her (without distractions or the worry of getting the house ready for visitors/ travelling anywhere!)”    “I feel the pandemic has overall positively affected my emotional relationship with my baby. It has led to uninterrupted time together where I am not distracted by coffee or classes. Although this would have at time provided me with light relief and distraction from lack of sleep(!) the rigid routine and undivided attention it has allowed me to give my baby seems to have made her very secure and happy.”    “Having to stay home has given us all an opportunity to relax into welcoming baby into our family. No rushing out in the morning for school run etc. Good quality time at home together. Aside from not being able to see family and friends in the flesh it's really not been that difficult for us. As a result I think my emotional relationship with my baby is as strong as anyone could wish for.”    “Husband was unhappy about returning to work after his two month paternity leave, but he has taken furlough since, and I on mat leave which has meant us enjoying lots of time together with the baby, and that has been very positive for all of us.”    “Having my partner home all the time has taken the pressure off of me, allowing me to rest, and take a small break when needed, which I think has positively affected my relationship with my baby.”    “It has been amazing to be able to share the first months of my baby's life with his dad and has really helped me to cope far better than with my first by sharing the load. I will find it very hard when he goes back to teaching and there are no resources available such as baby groups, meeting other mums, going on public transport to do things outside of walking distance from my house.” |
| Costs of Lockdown | 2. The burden of constant mothering | - Lack of practical support and childcare leading to the intensification of domestic and caregiving tasks - Increased feelings of exhaustion and guilt | “Not having childcare for my older child has meant that I could not concentrate in my new baby as much as I wanted. I am trying to carry out stimulating educational activities with my older child whilst in charge of cooking, washing, tidying up, breastfeeding, etc. both children are being affected and so am I. I am an exhausted mum unable to concentrate on either of my children and this is taking an [emotional] toll on everyone”  “I feel guilty I can’t give my baby the 1 to 1 time my other children got as babies as I have the other two home from school and nursery this is my main worry for him since lockdown”    “The thing I found most difficult during lockdown was full time care of my baby and my 3 year old daughter when I had planned that my older daughter would be in nursery 3 days a week and I would have some time to relax (&sleep!) during the nursery days, now nursery has restarted it is easier though now feel a bit more lonely/bored on those days.”    “We haven’t had anyone come over to give us a break since before lockdown and it’s exhausting. I miss visitors as I want to catch up with people properly but also they can take the baby for a few minutes so I can have a rest or catch up with housework etc.”    “It’s so full on. I can’t hand her over to my family for 30 minutes to have a shower. She’s bored and has stopped napping because she’s not tired enough as we can’t do much that’s engaging for her. I’m frustrated.”    “My new baby isn’t getting any one on one time with me, my eldest is 3 and nursery was closed two weeks after his birth. She is taking up the majority of my time and attention as is more demanding with her needs. The only time I can fully focus on my baby is when feeding.”    “Not having childcare for my older child has meant that I could not concentrate in my new baby as much as I wanted. I am trying to carry out stimulating educational activities with my older child whilst in charge of cooking, washing, tidying up, breastfeeding, etc. both children are being affected and so am I. I am an exhausted mum unable to concentrate on either of my children and this is taking an emoltional toll on everyone”    “I don’t get any time to just myself even if the baby is sleeping as the other kids are here all the time. It is relentless and exhausting. … It’s been a really difficult time, we’re trying to cope with my partner working from home, a new baby and three kids who would otherwise be at school and who are bouncing off the walls.”    “Not being able to share him with my friends and family means I often don’t have the energy to be as communicative with him as I would like. It’s a lot of pressure on one person when normally he would have engagement with others to balance things out.”    “I have been homeschooling my oldest child (5 years old) and he needs a lot of attention which has meant I haven’t been able to focus on my baby as much as I’d like. This has meant my relationship with both kids has been negatively affected. I’ve really struggled with this and [caring] for my baby.” |
|  | 3. Inadequacy of virtual contact | - The lack of “incidental support” with virtual contact - Lack of information transmission and affirmative support leading to low maternal confidence | “Only using social media is tough. Most of my friends and I don't talk on the phone we speak face to face so it's hard. I don't feel I can just call someone for a chat, especially as most are working from home or busy looking after their own kids. I'm so exhausted at the end of the day that I can't participate in zoom calls as I try to sleep early as my baby wakes every 2 hours.”  “I don't feel my family can make emotional connections with my son over video calls because he's too young.”    “The speed of these [telephone] appointments is great compared to the previous system of waiting for a doctors appointment, but you don't feel like they always necessarily see the whole picture without physical contact.”  “I have done a few zoom catch ups either for baby yoga or with friends to have a quiz. I find the zoom meetings exhausting and don't look forward to them which is strange because I am an extrovert and usually thrive when I see people.”    The cancellation or move of maternity / baby appointments to over the phone rather than face to face has resulted in increased concerns and anxiety as babies and mothers are not getting all the usual follow up checks, which when you also can't call on physical support of your network becomes even more worrying    “Have a premature baby and bringing her home during this time has been extremely difficult. Not having professionals see her in person has caused us a lot of anxiety in terms of confidence that she is healthy and we are doing a good job. … As a first time mother there is a huge amount of learning and many concerns and anxieties and a desperate need for reassurance and support that we have tried to replicate over the telephone but it is not the same.”    “By the time evening rolls round I'm so tired I don't have much energy for calls/texts etc but in the day time my hands are so full with the kids. It's much easier to interact with friends while also looking after a baby if you are face to face whereas it doesn't really work well on a screen.”    “I am someone who has a history of [anxiety and depression] and struggled a lot in her first month. I think I would have benefitted from professional support but felt unable to ask. I wonder if my appointments with health professionals had been face to face the might have better picked up on cues and made it easier for me to access this. I felt I was given a standard ‘how are you feeling’ question by my health visitor I was too scared to answer honestly.”    “The situation has been [hard] because [I] can't: access support groups physically See my friends See my family risk free See health practitioners easily. The home care and support from key workers has been virtual and not overly helpful.”  “It has definitely made me more anxious - am I doing enough for my baby, is she ok, is she healthy and happy, should I be doing more, do people think I'm a good mother. Much of this is because it's hard to communicate online and people aren't able to see how I'm coping or share in the joy of my baby.” |
|  | 4. Mourning lost opportunities | - Inability to expand mother-infant social networks - Feelings of sadness for lost experiences, for both mum and baby - Worries about baby’s development due to lack of physical contact with others | “I feel sadness that this special time has be taken away from me. Although I have bonded with my baby I feel robbed of all the social aspects of being a mummy eg baby classes, interaction with other mums and babies. I'm upset that once this pandemic is over, it will be time for me to return to work. It makes me extremely sad and frustrated”    “I feel my year of maternity leave where I was meant to make new friends with other mums and ensure my baby develops socially has been stolen from me. I get one year to spend with my baby giving her the best start in life and I feel I can no longer do that as instead, we are at home all day bored. All the pressure is on me and I miss my friends and life I thought I was going to have this year.”    I feel sadness that this special time has be taken away from me. Although I have bonded with my baby I feel robbed of all the social aspects of being a mummy eg baby classes, interaction with other mums and babies. I'm upset that once this pandemic is over, it will be time for me to return to work. It makes me extremely sad and frustrated”    “[I] was looking forward to certain things on maternity leave such as meeting up with friends, going to cinema with my baby, art galleries etc, have sort of had to mourn the loss of my ideas of how maternity leave would be as [we] do not plan to have any more children so this is my last maternity leave”    “I feel sad that he's not been able to get out and about and have the social interactions due to the pandemic. Feels like lost time to bond with friends and family and see things outside of our house.”    “I worry that my child isn't getting the development they deserve as they are not getting enough stimulus.”    “I have found it difficult to not be able to go out with my baby and meet other mother's. I feel so guilty that he will not be able to play with other children and worry how it will affect him.”    “I'm worried he will miss out on socialising elements as he has only met grand parents once since being born and not been held by anyone apart from my husband in the last 10 weeks.”    “My main worry is the impact social distancing will have on my [son’s] relationships with other people. Currently he only [knows] me & my husband. I worry about him not being socialised / becoming clingy”    “I am worried about the developmental impact this has had on the baby - will she be able to cope with meeting other people / being held by them etc. Will her separation anxiety be worse than usual.”    “I don't know anyone with a new baby as NCT dried up and I cannot go to any groups or meet anyone. Friends and family visit/ speak on video chats but I am missing spending time with other mums. I don't know how that will impact me or my baby long term.”  “I also feel really sad that she's growing up so fast without getting to spend time with many of the people we love the most (Skype etc is a poor substitute for face to face).”  “Every day, I spend 12 hours on my own with no other contact until [my husband] comes home. If times were ‘normal’, I'd be seeing other family and my NCT group but instead I'm physically isolated from everyone which in turn has led to decreased virtual contact too.”  “I feel my year of maternity leave where I was meant to make new friends with other mums and ensure my baby develops socially has been stolen from me. I get one year to spend with my baby giving her the best start in life and I feel I can no longer do that as instead, we are at home all day bored. All the pressure is on me and I miss my friends and life I thought I was going to have this year.” |
