# Supplementary figures and images for: Communication Across Maternal Social Networks During England’s First National Lockdown and Its Association With Postnatal Depressive Symptoms
Source: Front Psychol. 2021 May 11;12:648002. doi: 10.3389/fpsyg.2021.648002 (PMC8144711; doi:10.3389/fpsyg.2021.648002)

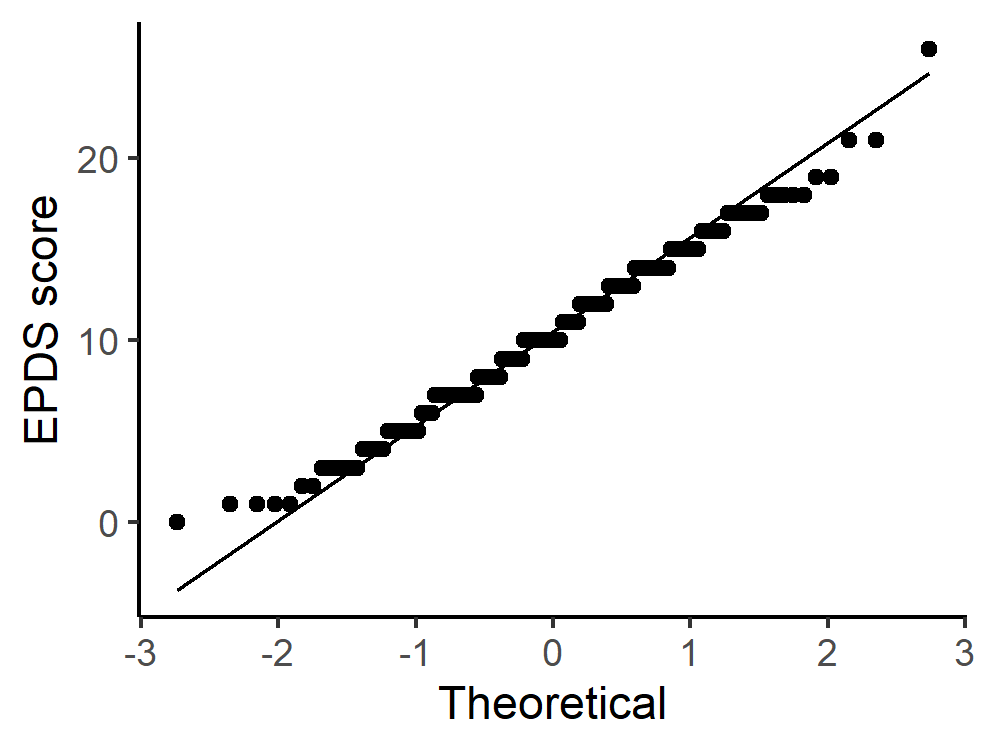

Supplement: Image 1.TIFF — ‘Supplementary Figure 1’ [file Image_1.TIFF]

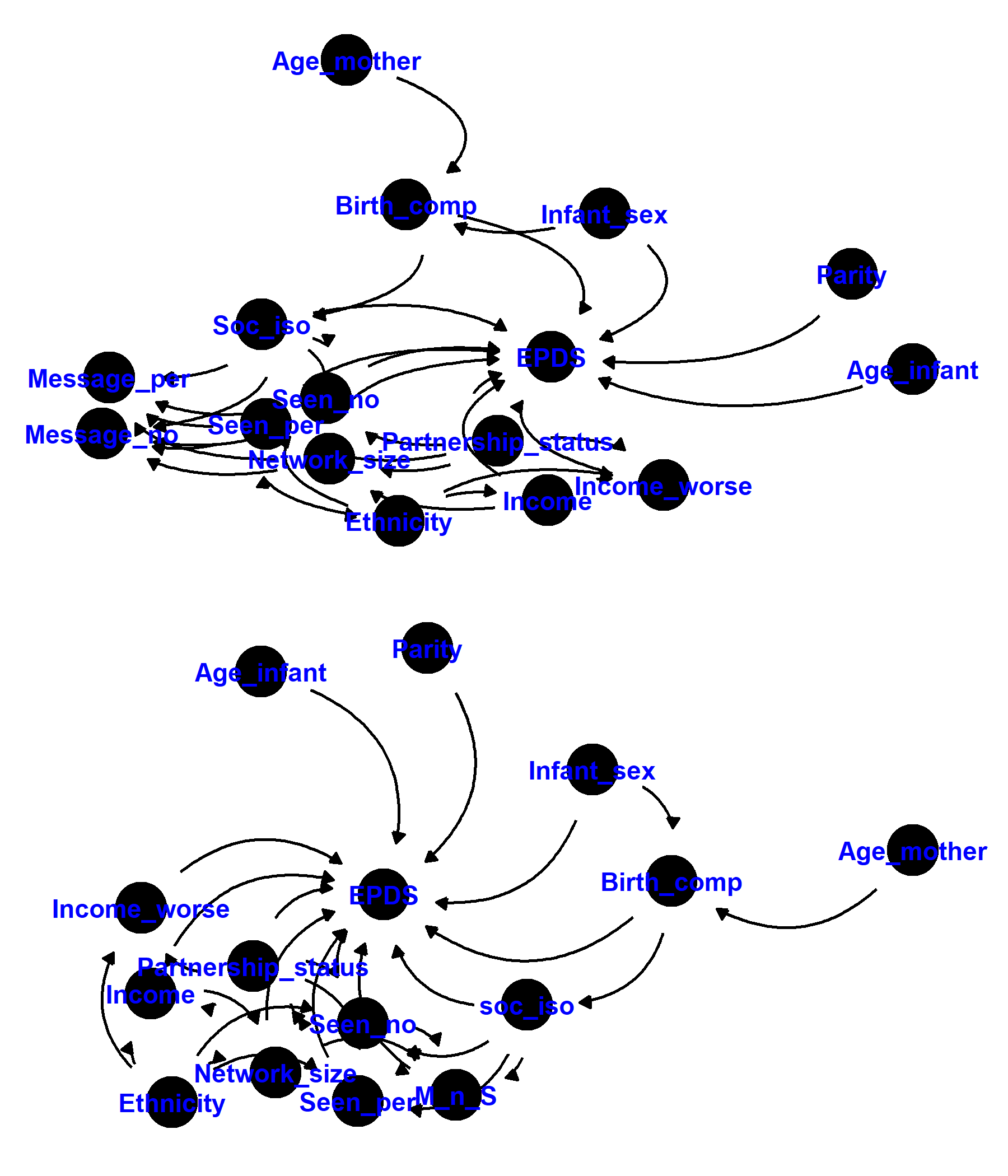

Supplement: Image 2.TIFF — ‘Supplementary Figure 2’ [file Image_2.TIFF]

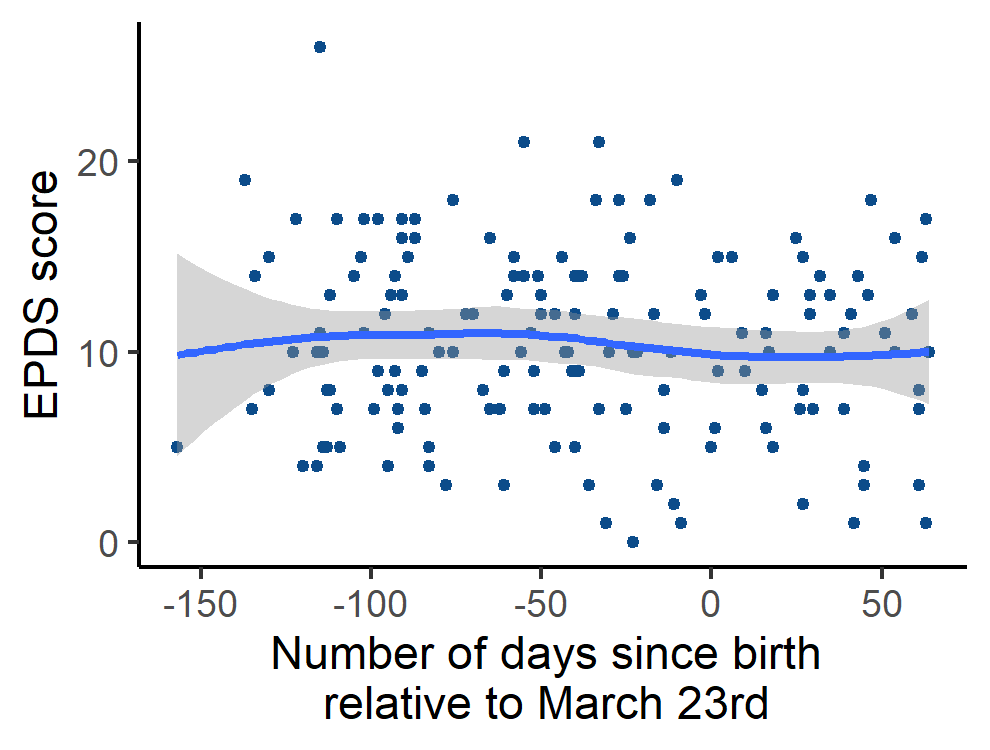

Supplement: Image 3.TIFF — ‘Supplementary Figure 3’ [file Image_3.TIFF]

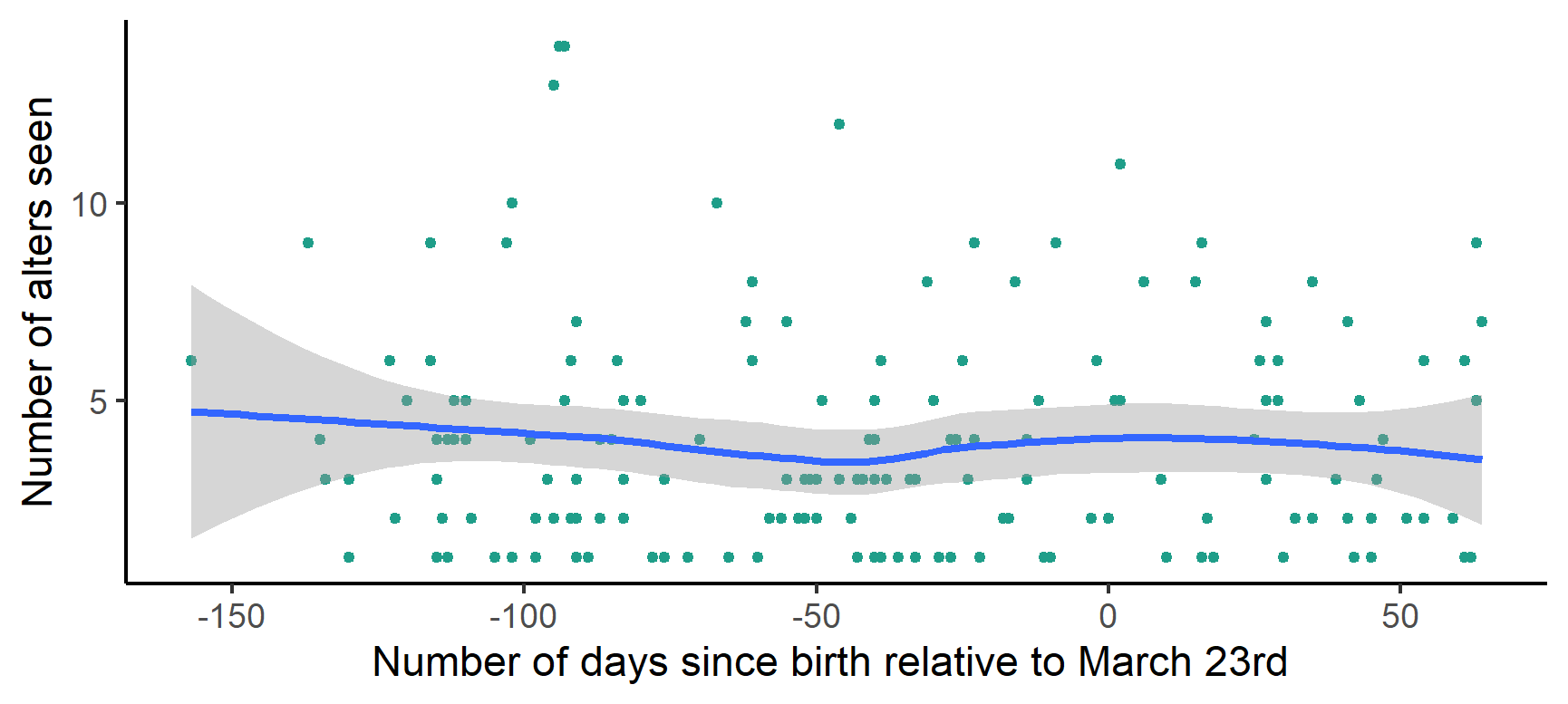

Supplement: Image 4.TIFF — ‘Supplementary Figure 4’ [file Image_4.TIFF]

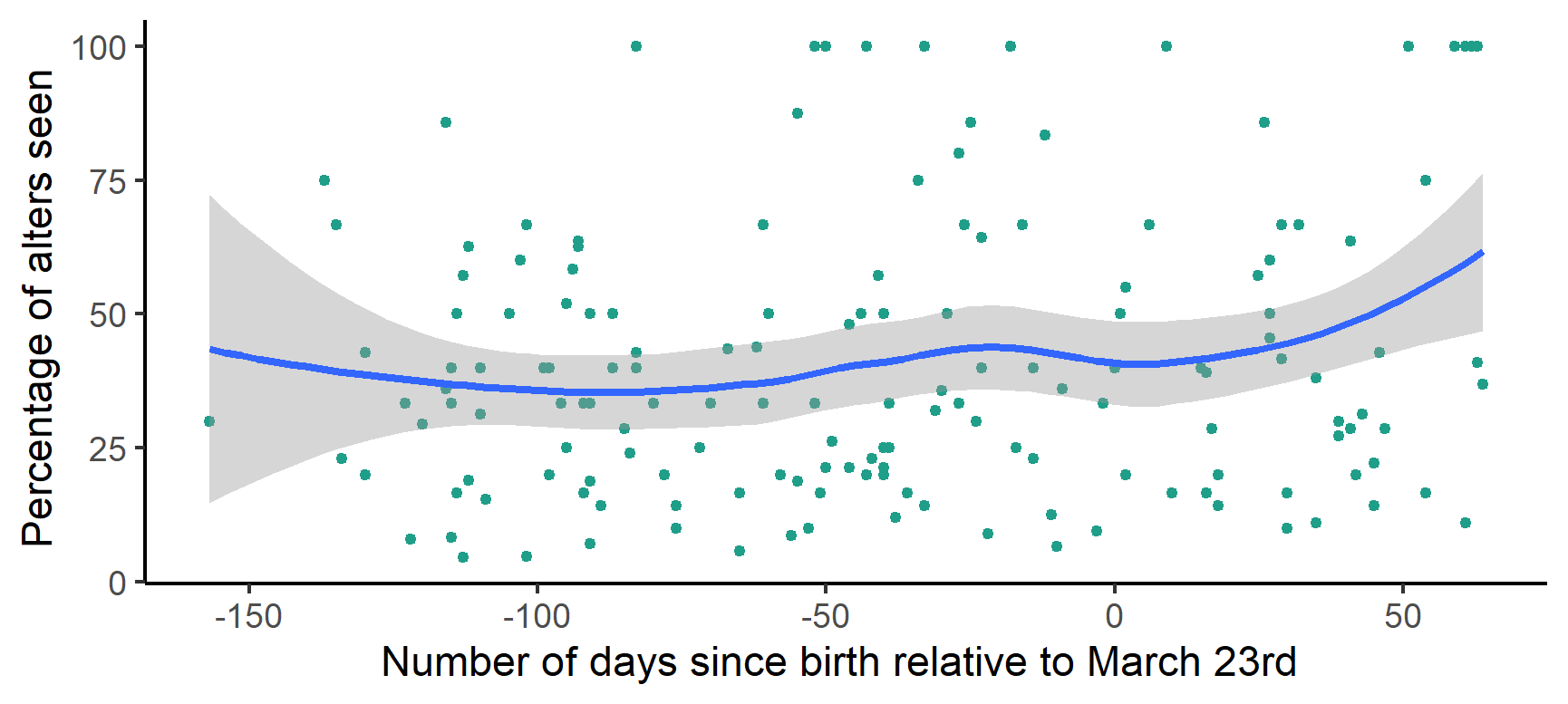

Supplement: Image 5.TIFF — ‘Supplementary Figure 5’ [file Image_5.TIFF]

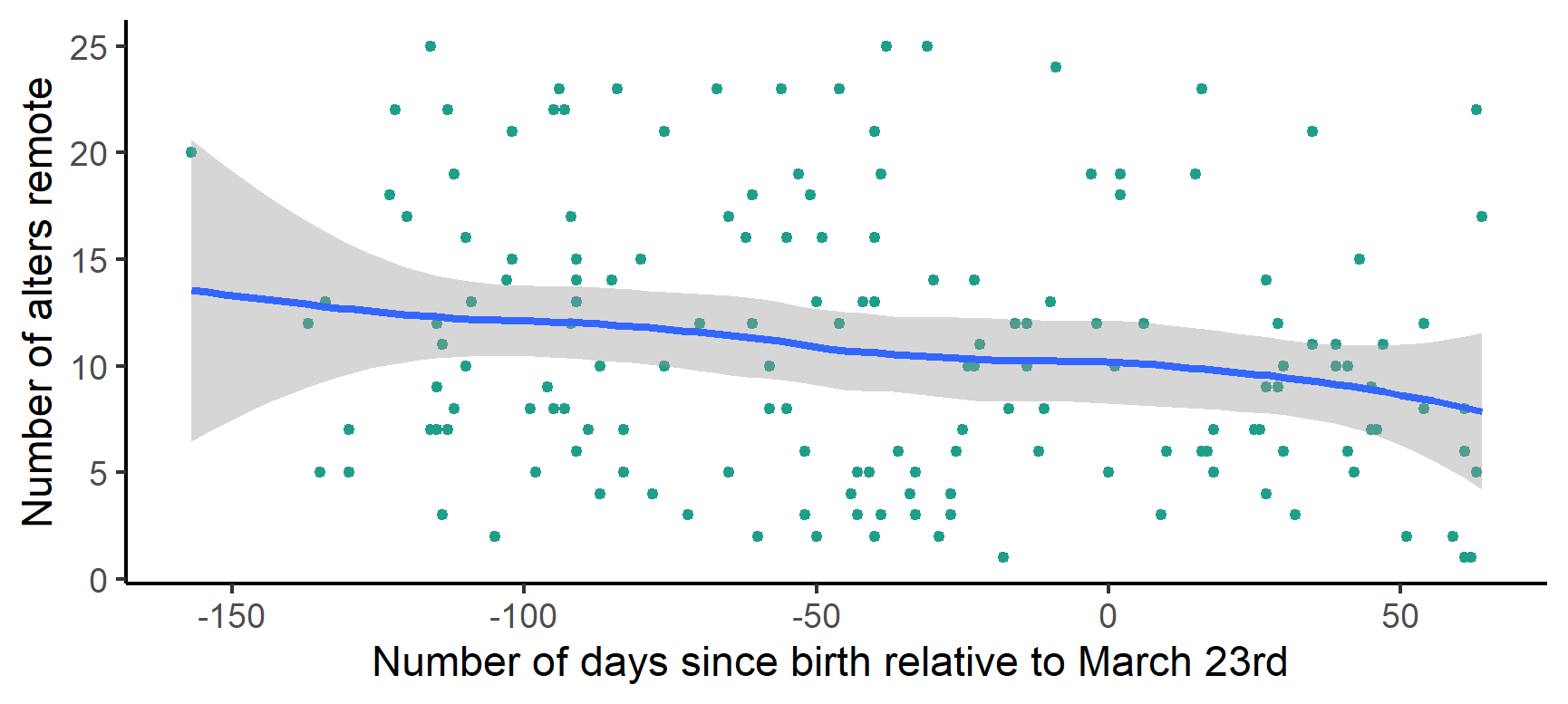

Supplement: Image 6.TIFF — ‘Supplementary Figure 6’ [file Image_6.TIFF]

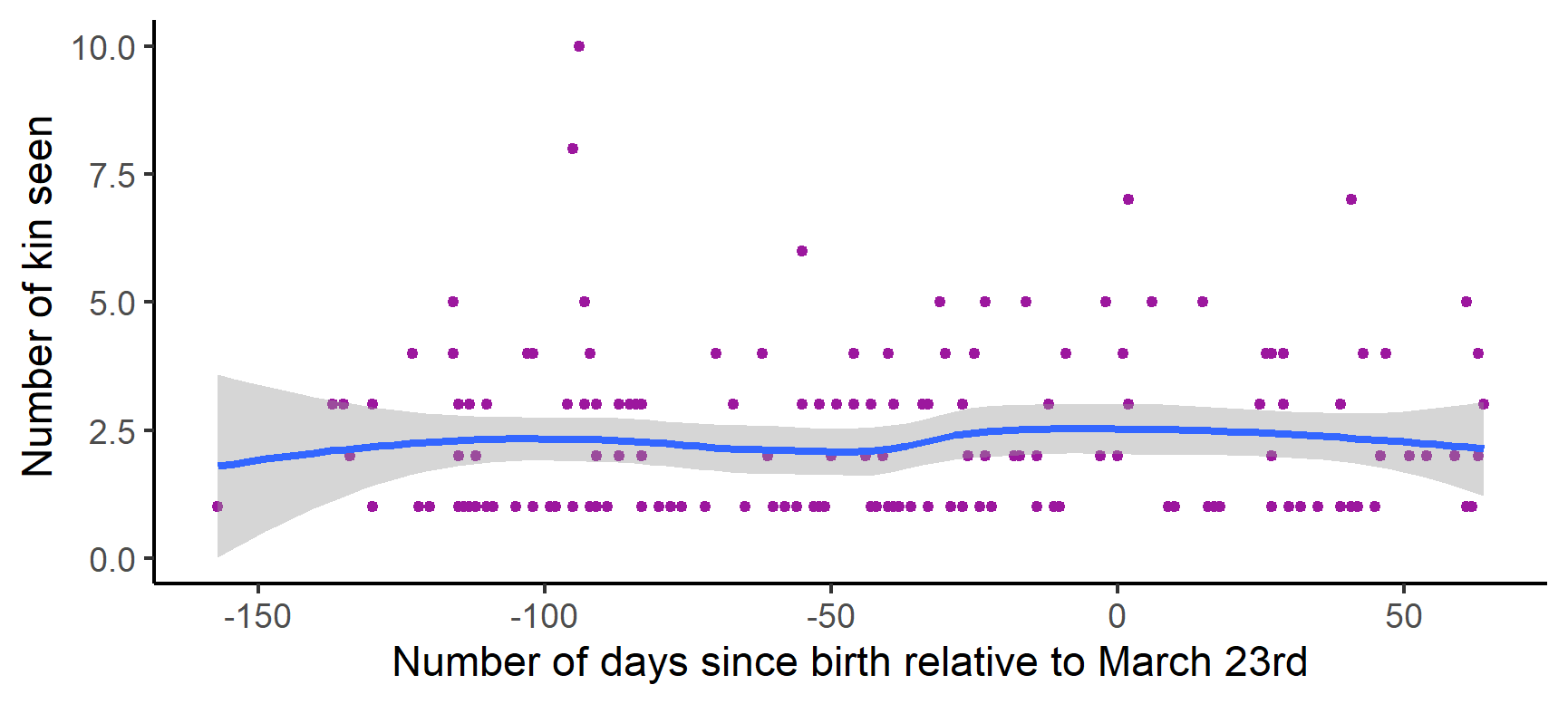

Supplement: Image 7.TIFF — ‘Supplementary Figure 7’ [file Image_7.TIFF]

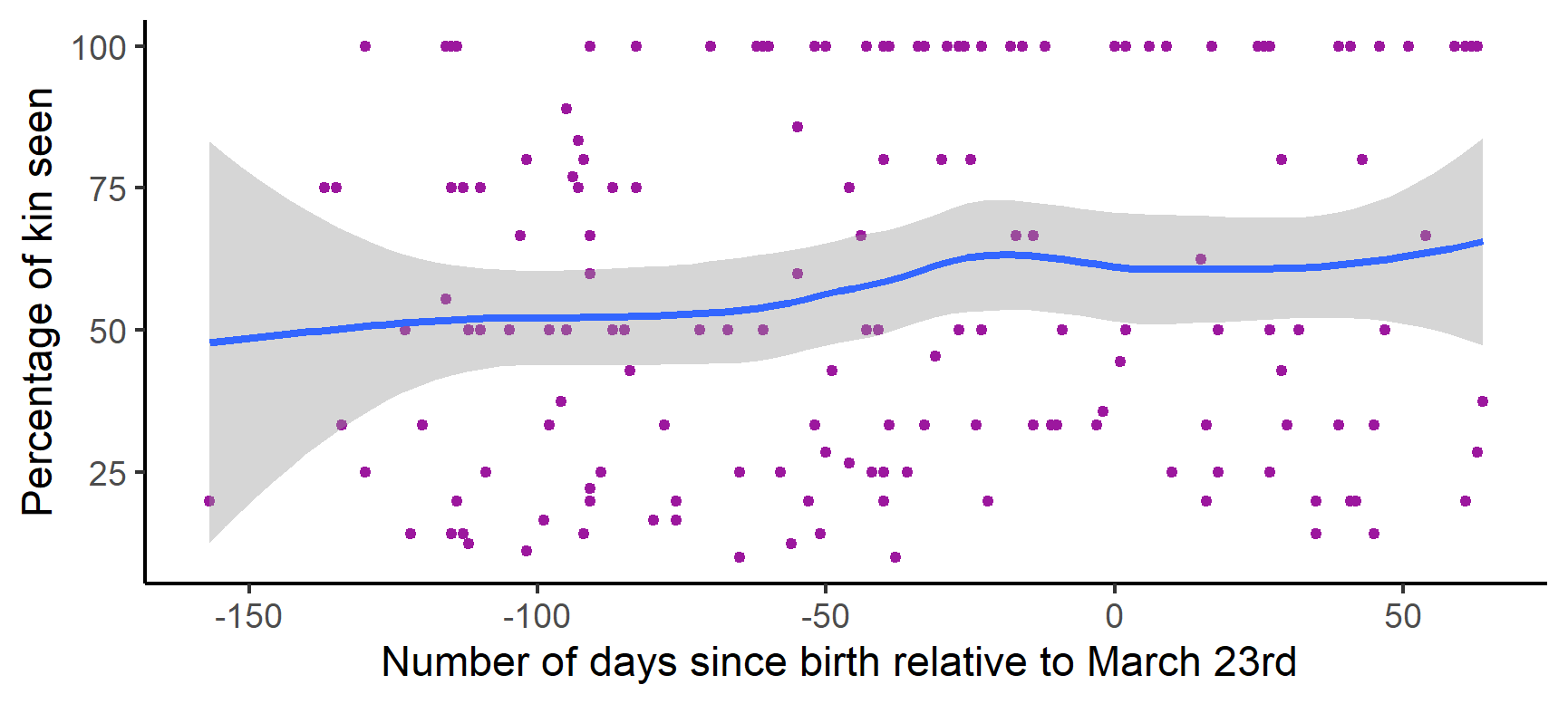

Supplement: Image 8.TIFF — ‘Supplementary Figure 8’ [file Image_8.TIFF]

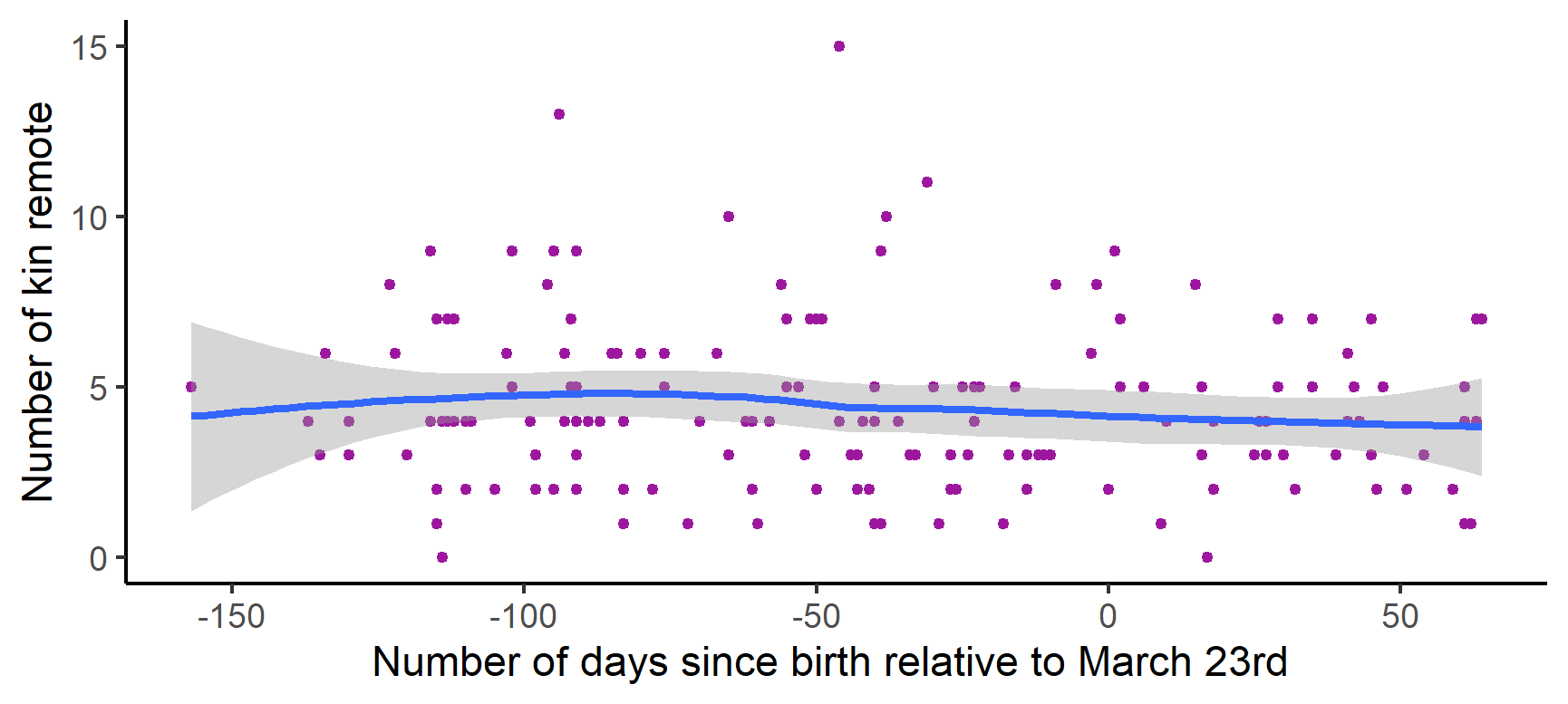

Supplement: Image 9.TIFF — ‘Supplementary Figure 9’ [file Image_9.TIFF]

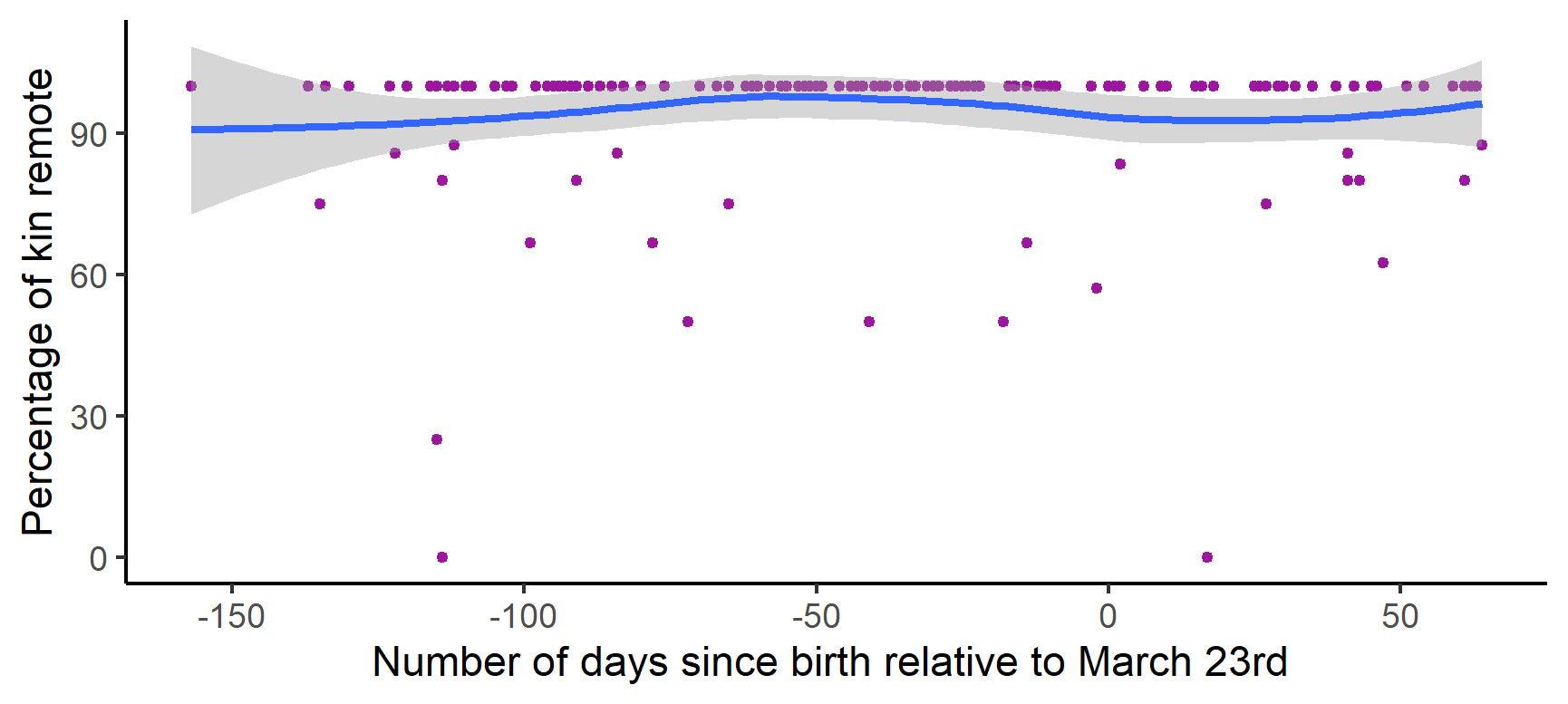

Supplement: Image 10.TIFF — ‘Supplementary Figure 10’ [file Image_10.TIFF]

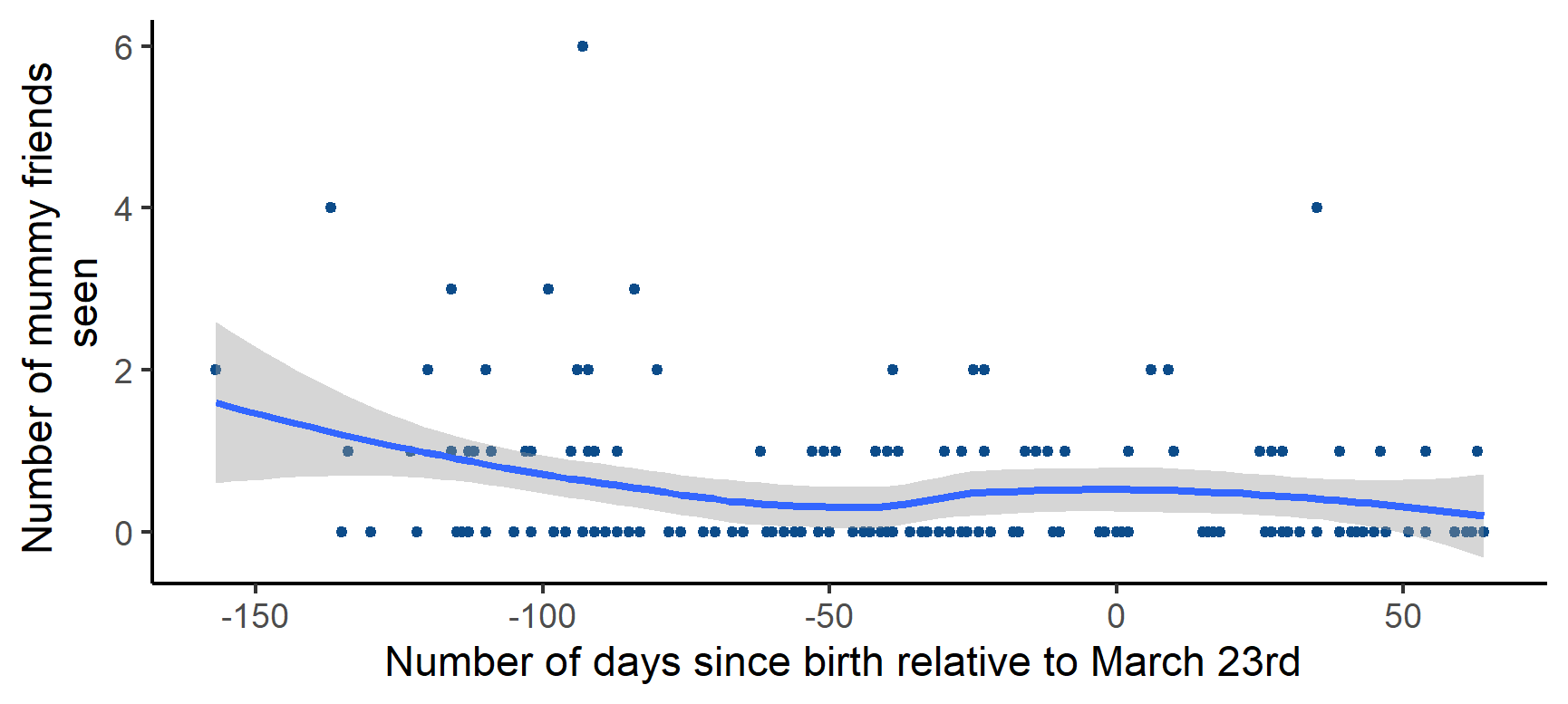

Supplement: Image 11.TIFF — ‘Supplementary Figure 11’ [file Image_11.TIFF]

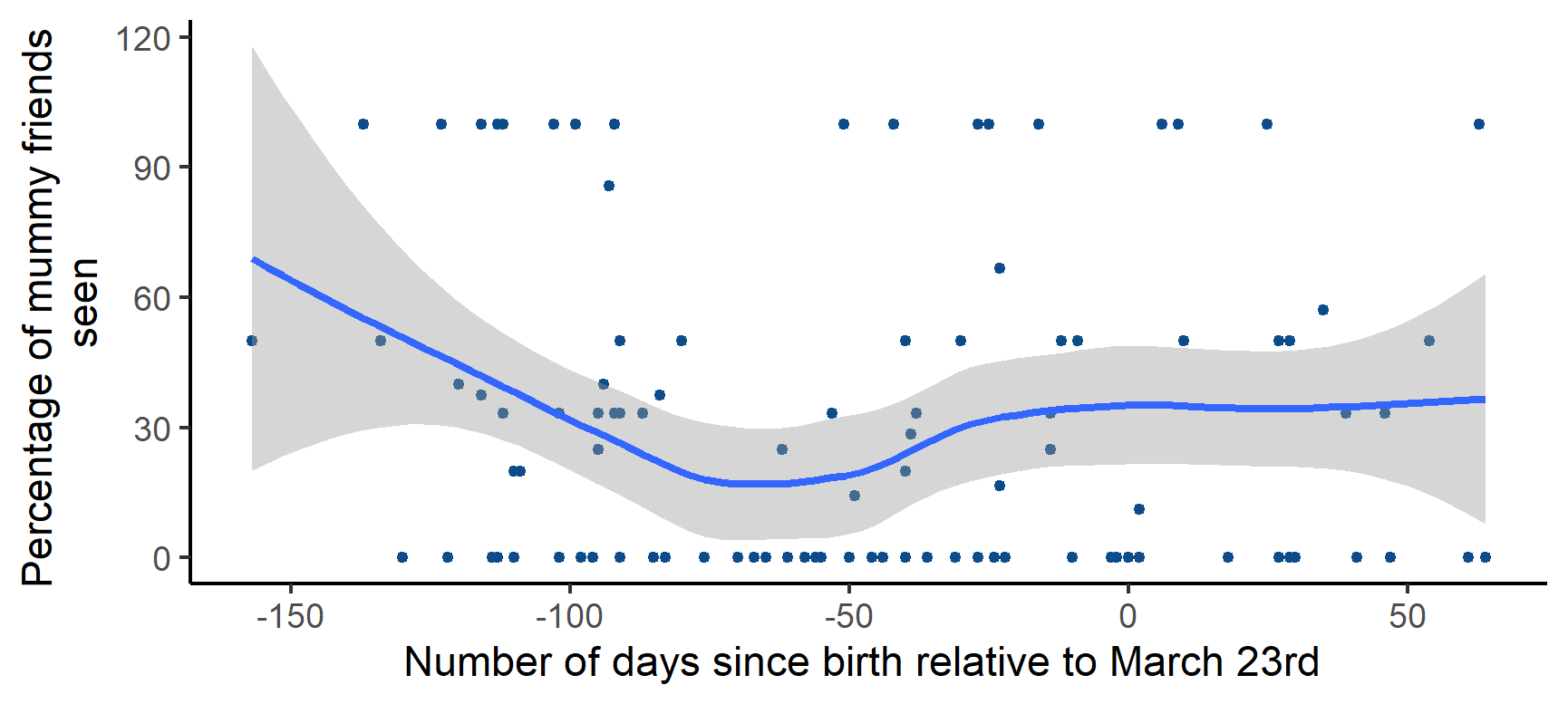

Supplement: Image 12.TIFF — ‘Supplementary Figure 12’ [file Image_12.TIFF]

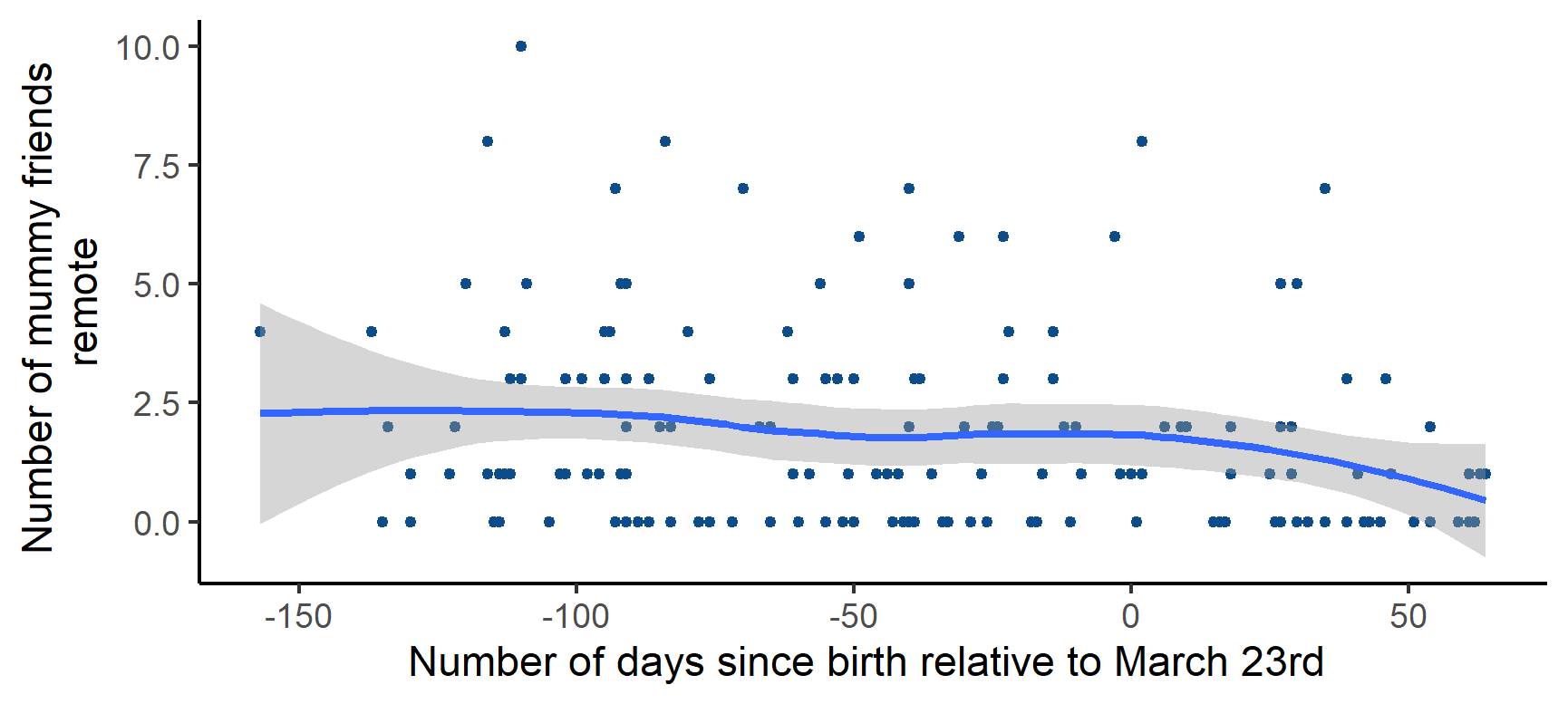

Supplement: Image 13.TIFF — ‘Supplementary Figure 13’ [file Image_13.TIFF]

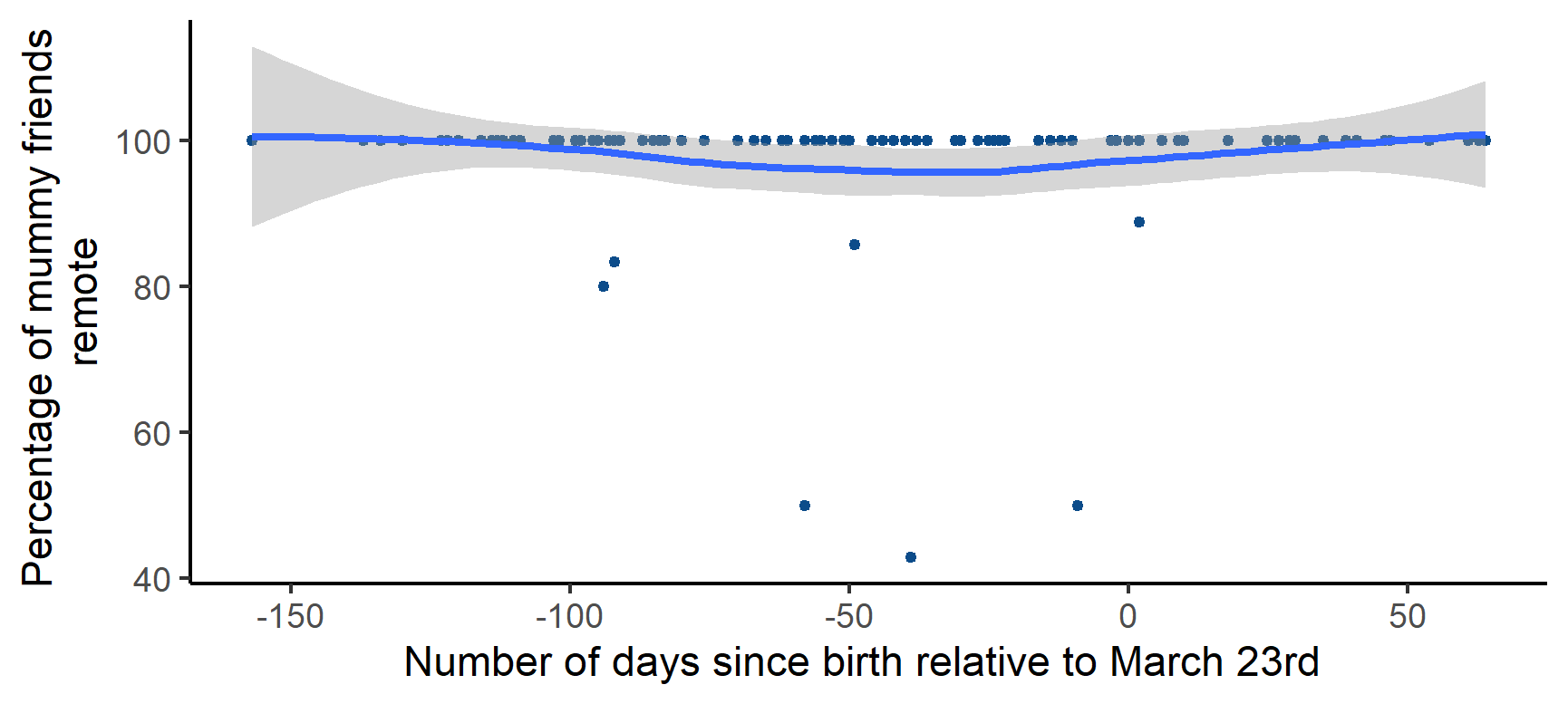

Supplement: Image 14.TIFF — ‘Supplementary Figure 14’ [file Image_14.TIFF]

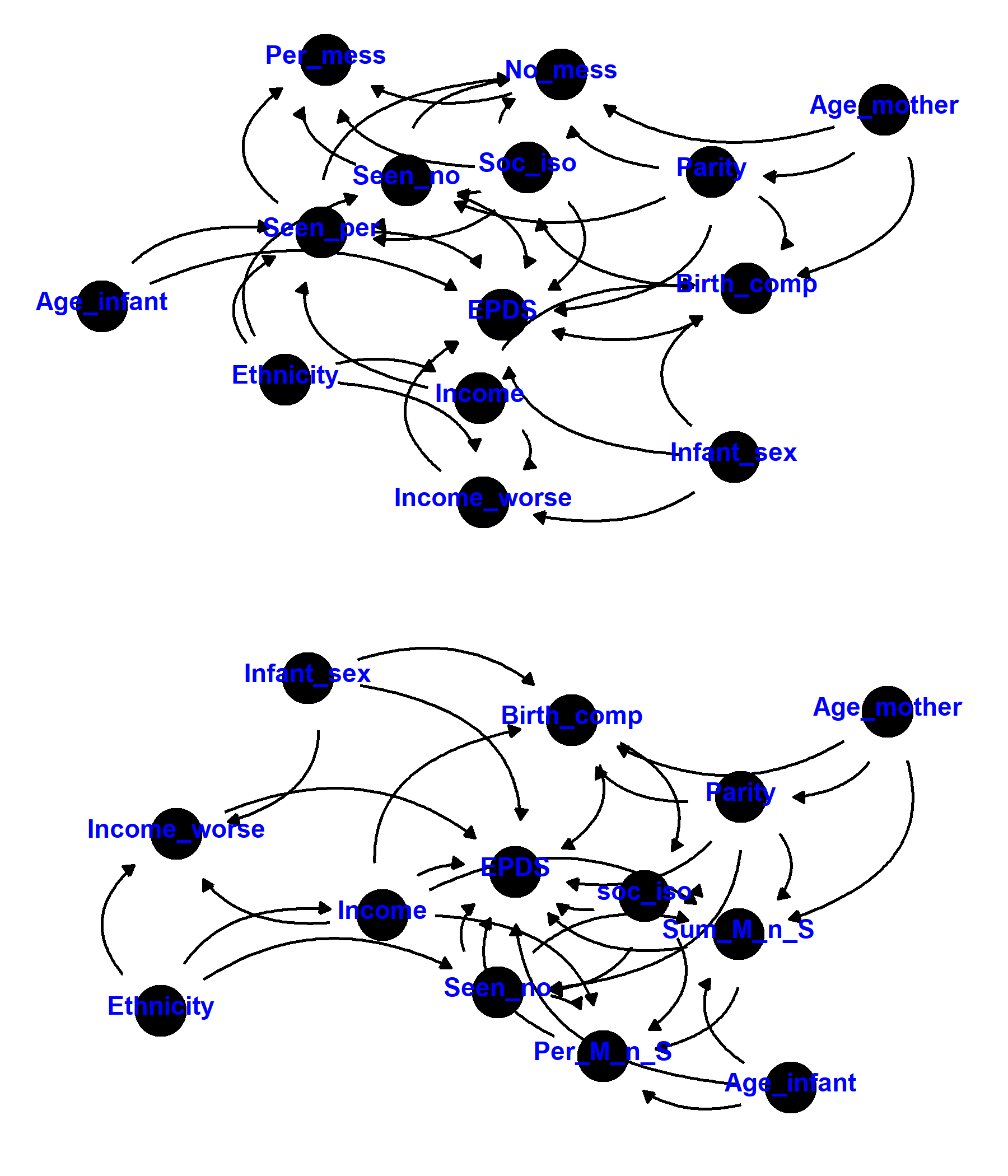

Supplement: Image 15.TIFF — ‘Supplementary Figure 15’ [file Image_15.TIFF]
